# Genome-Wide Characterization of a Highly Penetrant Form of Hyperlipoprotein(a)emia Associated With Genetically Elevated Cardiovascular Risk

**DOI:** 10.1161/CIRCGEN.121.003489

**Published:** 2022-02-07

**Authors:** Stefan Coassin, Kevin Chemello, Ilya Khantalin, Lukas Forer, Patricia Döttelmayer, Sebastian Schönherr, Rebecca Grüneis, Clément Chong-Hong-Fong, Brice Nativel, Stéphane Ramin-Mangata, Antonio Gallo, Mathias Roche, Beatrix Muelegger, Christian Gieger, Annette Peters, Johannes Zschocke, Catherine Marimoutou, Olivier Meilhac, Claudia Lamina, Florian Kronenberg, Valentin Blanchard, Gilles Lambert

**Affiliations:** Institute of Genetic Epidemiology, Department of Genetics and Pharmacology (S.C., L.F., P.D., S.S., R.G., C.L., F.K.), Medical University of Innsbruck, Austria.; Institute of Human Genetics (B.M., J.S.), Medical University of Innsbruck, Austria.; Université de La Réunion, INSERM UMR 1188 DéTROI, Sainte-Clotilde, France (K.C., I.K., C.C.-H.-F., B.N., S.R.-M., A.G., M.R., O.M., V.B., G.L.).; CHU de La Réunion, Service de Chirurgie Cardiaque Vasculaire et Thoracique, Saint-Denis, France (I.K.).; Research Unit of Molecular Epidemiology (C.G.), Helmholtz Zentrum München, German Research Center for Environmental Health, Neuherberg, Germany.; Institute of Epidemiology (C.G., A.P.), Helmholtz Zentrum München, German Research Center for Environmental Health, Neuherberg, Germany.; German Center for Diabetes Research (DZD), München-Neuherberg, Germany (C.G., A.P.).; CHU de La Réunion, CIC EC1410, Saint-Pierre, France (C.M., O.M.).; Department of Medicine, Centre for Heart Lung Innovation, Providence Healthcare Research Institute, St Paul’s Hospital, University of British Columbia, Vancouver, Canada (V.B.).

**Keywords:** apolipoproteins, coronary artery disease, lipids, lipoprotein(a), risk factors

## Abstract

Lp(a) (lipoprotein [a]) is a highly atherogenic lipoprotein strongly associated with coronary artery disease (CAD). Lp(a) concentrations are chiefly determined genetically. Investigation of large pedigrees with extreme Lp(a) using modern whole-genome approaches may unravel the genetic determinants underpinning this pathological phenotype.

**Methods:**

A large family characterized by high Lp(a) and increased CAD incidence was recruited by cascade screening. Plasma lipids, lipoproteins, and apolipoproteins concentrations, as well as the size of apo(a) isoforms, were determined enzymatically by high-resolution mass spectrometry and Western blot, respectively. Whole-exome sequencing was performed to search for rare defects in modifier genes. Genetic risk scores (GRS) for Lp(a) and CAD were calculated and their discriminative power was assessed.

**Results:**

Seventeen individuals displayed extreme Lp(a) levels including 6 with CAD. Whole-exome sequencing showed no hint for genetic defects outside the *LPA* locus. The extreme Lp(a) phenotype segregated with the presence of a short apo(a) isoform containing 21 Kringle IV domains. This allele was characterized by the presence of three rare strongly Lp(a) increasing single nucleotide polymorphisms and a significantly increased load of oxidized phospholipids per Lp(a) particle. An Lp(a) GRS consisting of 48 single nucleotide polymorphisms that represent 2001 genome-wide significant *LPA* single nucleotide polymorphisms, efficiently captured the hyper-Lp(a) phenotype and discriminated affected and nonaffected individuals with great accuracy. The genome-wide GRS for CAD, encompassing 6.6 million single nucleotide polymorphisms, was very high for most family members (>97.5 percentile of the reference population), but this observation was no longer valid when the contribution of the *LPA* locus was omitted.

**Conclusions:**

High-Lp(a) phenotypes can be successfully captured using the Lp(a) GRS even among closely related family members. In hyper-Lp(a) individuals, *LPA* can be a major locus driving a very high CAD GRS. This underpins the large contribution of the *LPA* locus to the cardiovascular genetic risk in families.

**See Editorial by Langsted and Nordestgaard**

Lp(a) (lipoprotein [a]) is a highly atherogenic lipoprotein causatively, independently, and significantly associated with cardiovascular diseases and calcified aortic valve stenosis.^1,2^ The major structural difference between Lp(a) and LDL (low-density lipoproteins) is that Lp(a) contains a unique signature protein, apolipoprotein(a) (apo[a]) covalently linked to apo B_100_.^3^ The atherogenicity of Lp(a) does not only stem from its LDL moiety rich in cholesterol but also because it is a sink for oxidized phospholipids (oxPL).^3,4^

Apo(a) is the product of the *LPA* gene located on chromosome 6q26-27.^1^ It presents a highly repetitive structure consisting of 10 subtypes of the plasminogen-derived KIV (kringle IV) domains (KIV-1 to KIV-10), followed by one kringle V and one inactive protease domains. Two enhancer regions (named DH-II and DH-III [DNase Hypersensitive sites II and III]) have been identified ≈20 to 30 kb upstream of *LPA*,^5^ and the promoter region extends for at least 4 kb upstream of *LPA*.^6,7^ The KIV-2 domain is encoded by a pair of exons that can be repeated 1 to 40 times per allele.^8^ The major consequence of this copy number variation is that the size of apo(a) is highly polymorphic, its molecular weight ranging from 300 to 800 kDa. Apo(a) isoforms size is inversely correlated with plasma Lp(a) concentrations, as it correlates with endoplasmic reticulum retention time, and explains 30% to 70% of Lp(a) variability.^1^ Overall, the whole *LPA* locus explains up to 90% of Lp(a) variability,^9^ indicating that additional strong modulators of Lp(a) concentrations reside within the *LPA* locus and account for the fact that Lp(a) levels can vary by 200-fold even among carriers of apo(a) isoforms of identical sizes.^10^

In line with this, same-sized *LPA* alleles still differ in the haplotypes of the single nucleotide polymorphisms (SNPs) they carry.^11,12^ For instance, SNPs rs10455872 and rs3798220 are largely used in the field, as they allow partial tagging of small apo(a) isoforms.^1,13^ In addition, specific SNP haplotypes associate with Lp(a) concentrations that can be much lower or much higher than what would be expected from the sole size of the isoforms.^11,12^

Some examples of such variants have been reported,^14–16^ but many more probably exist. Genome-wide association studies (GWAS) have identified hundreds of variants associated with Lp(a) levels.^17,18^ A majority of these variants are distributed over a ≈2 megabases region around the *LPA* locus,^17,18^ but causality for modulating Lp(a) levels has been established only for a handful. The important contribution of additional SNPs to Lp(a) concentration is also illustrated by the fact that within families, same-sized apo(a) isoforms are associated with a much narrower Lp(a) variability (2- to 3-fold) than in the general population.^10^ The occurrence of high *LPA* expressing alleles can thus confer a highly penetrant cardiovascular risk to individuals and families,^19–22^ similar to what is seen in familial hypercholesterolemia.^23,24^

Although *LPA* is the major determinant of Lp(a) in the population, it is unclear whether rare defects in other genes^25^ can also be at the origin of some hyper-Lp(a) phenotypes. Investigations of pedigrees with extreme phenotypes using modern whole-genome technologies might help unravel the genetic determinants of hyperlipoprotein(a)emia. To test this possibility, we have undertaken a comprehensive investigation of a unique pedigree recruited through cascade screening from an individual with no cardiovascular risk factors other than an extreme Lp(a) concentration who underwent recurrent coronary syndromes,^20^ using whole-exome sequencing, targeted analysis of the *LPA* locus, genetic risk score (GRS) computation for Lp(a) and coronary artery disease (CAD), as well as extensive biochemical assessment of their Lp(a).

## Methods

Detailed methods are available in the Supplemental Methods and Tables S1 through S5 in the Supplemental Material. Ethics approval was granted by the Comité de Protection des Personnes Sud Méditerranée (ID: 2020-A00196-33). Informed consent was obtained from all participants, and all studies were performed in accordance with the Declaration of Helsinki. All participants gave written informed consent for genetic testing and research. The data that support the findings of this study are available from the corresponding author upon reasonable request.

## Results

### Unique Multigenerational Pedigree

**Figure 1.**
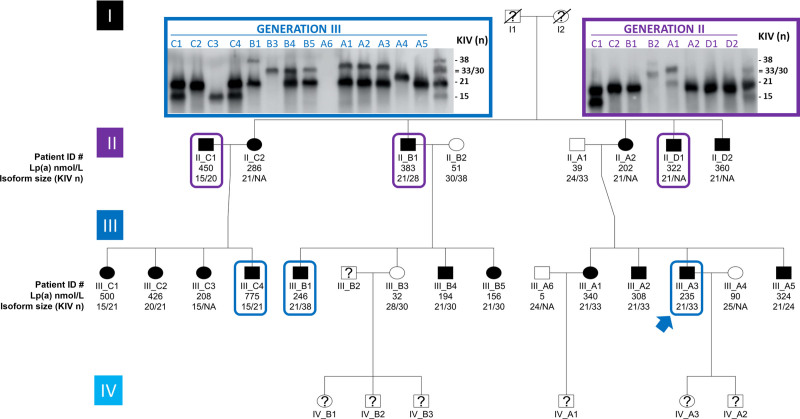
**Pedigree of the family.** Twenty-two individuals (17 related family members and 5 spouses) were recruited by cascade screening through the index patient (blue arrow). Black symbols denote a high-Lp(a) (lipoprotein [a]) phenotype (>150 nmol/L) and white symbols normal Lp(a) phenotypes (≤90 nmol/L). Below each symbol, the first line displays individual ID numbers, the second line plasma Lp(a) concentrations (in nmol/L), and the third line the size of their apo(a) isoforms (number of KIV [kringle IV] domains). Individuals with established premature coronary artery disease are framed. Not avialable, as isoform expression is too low for detection. For each individual (n=22), plasma lipids and lipoproteins concentrations, including Lp(a) were assessed in plasma samples at least three times independently. Western blots used to determine apo(a) isoform sizes were performed twice on each plasma sample in 2 independent experiments. A representative Western Blot is displayed (inset).

Seventeen related family members and five spouses were recruited through the index patient (III-A3) by cascade screening (Figure 1, Table S1 in the Supplemental Material). The 17 relatives descend from unrelated grandparents from La Réunion Island. The grandfather (I-1), who was a heavy smoker, had myocardial infarction (MI) at age 60 years and died from lung cancer at 80 years. The grandmother (I-2) died at 82 from recurrent episodes of MI and stroke. Among their 5 children (3 males/2 females), 2 sons (II-B1 and II-D1) had MI at ages 52 and 50 years, respectively. One was and still is overweight (body mass index, 29.4 kg/m^2^) and both smoked. One daughter (II-C2), also overweight (body mass index, 26.9 kg/m^2^), developed diabetes in her forties. Her husband (II-C1) had severe MI at 38 and also presented with type 2 diabetes (body mass index, 26.4 kg/m^2^) as well as elevated cholesterol levels (5.79 mmol/L).

Among the 14 family members of the third generation, in addition to the index case patient (III-A3) who had recurrent MI episodes at age 32 years,^20^ two of his male cousins had severe MI at ages 27 (III-C4) and 35 (III-B1), respectively. The first smoked occasionally and had slightly elevated total cholesterol (6.52 mmol/L) and triglycerides (2.20 mmol/L). The second was hypertensive (systolic blood pressure/diastolic blood pressure 150/90 mm Hg) and overweight (body mass index, 29.3 kg/m^2^). Among the 16 family members who have not developed any cardiovascular event yet, 8 were overweight, 3 were hypertensive, and 1 smoked. Seven had elevated total cholesterol levels (>5 mmol/L). Not a single family member displayed impaired renal function or aortic valve stenosis. Strikingly, plasma Lp(a) concentrations were found above the threshold of 125 nmol/L (ranging from 156 to 775 nmol/L) in all but one (III-B3) relatives as well in one out of 5 spouses (II-C1).

Noteworthy, LDL-C (LDL-cholesterol) levels were on average undistinguishable between family members with or without Lp(a) above 125 nmol/L (3.03±0.73 versus 3.00±0.78 mmol/L). When LDL-C values were corrected for Lp(a) cholesterol (assuming a cholesterol content in Lp[a] of 30%), there was a trend for lower corrected LDL-C levels in family members with Lp(a) above 125 nmol/L (1.94±0.90 versus 2.86±0.99 mmol/L, respectively, *P*=0.064; Table S1 in the Supplemental Material). Most individuals in this large pedigree, therefore, display hyperlipoprotein(a)emia without true elevated LDL-C levels.

### Whole-Exome Sequencing

We first hypothesized that a yet unidentified putative or regulator defect might cause the extraordinarily high-Lp(a) concentrations observed in a majority of pedigree members. Although anecdotal reports about hyper-Lp(a) pedigrees are known in the field, a comprehensive genetic evaluation of such a pedigree has not been performed before. We thus performed whole-exome sequencing in 13 family members (9 with high Lp[a]) (the other pedigree members joined the study after completion of this analysis). This yielded 316 251 SNPs and 52 676 indels. Ninety-one thousand six hundred sixty-two SNPs and 6709 variants were retained after filtering for a minimum coverage of 4× and localization within ±50 base pairs from any exon annotated in National Center for Biotechnology Information Reference Sequence Database Release 105. Seventeen SNPs in 13 genes (8 missense variants, seven 3′ untranslated region SNPs, 2 5′ untranslated region SNPs) and no indels segregated exclusively with the high-Lp(a) phenotype (assuming a dominant mode of inheritance; Table S6 in the Supplemental Material). None of these genes except *LPA* has any known connection to lipid metabolism and/or were plausible candidates (Supplemental Notes). Also, relaxation of filtering parameters by allowing for one individual being a phenocopy or up to 3 individuals being also homozygous for causal variants (in case the causal variant is unexpectedly frequent) as well as manual inspection of the candidate Lp(a) receptors recently reported^26^ did not reveal additional candidate variants (Table S7 in the Supplemental Material, Supplemental Notes).

Taken together, whole-exome sequencing data found no clear hint for a receptor defect, which was also suggested by similar cellular uptake of Lp(a) observed in lymphocytes isolated from family members with high versus normal Lp(a) phenotypes (Supplemental Results and Figure S1 in the Supplemental Material).

### LPA Gene Locus

Elevated Lp(a) levels were systematically associated with the presence of one apo(a) isoform containing 21 KIV domains in this family, except for 2 individuals: II-C1 who entered the pedigree by marriage and presents another isoform combination associated with high Lp(a) (15 KIV and 20 KIV) as well as his daughter III-C3 who inherited the 15 KIV allele (Figure 1). The high expressing 21 KIV allele was characterized by concomitant occurrence of the rare *LPA* SNPs rs3798220 (protease domain), rs186696265 (enhancer region; ≈26 kb upstream of *LPA*), and rs140570886 (KIV-6, intronic; Tables S6, S8, and S9 in the Supplemental Material). Among carriers of that allele, the expression of the 21 KIV isoform was predominant, accounting on average for 86±14% of total Lp(a) (Table S9 in the Supplemental Material). The rare variants rs186696265 and rs140570886 were the strongest Lp(a) increasing SNPs in a recent GWAS.^17^ The *LPA* allele with 21 KIV of individual II-A2 was isolated by long-range pulsed-field gel electrophoresis and the *LPA* enhancer region was subjected to Sanger sequencing. This confirmed that rs186696265 is located on the 21 KIV allele (Table S8 in the Supplemental Material). Phased genotypes from imputation indicate that the minor alleles of rs3798220 and rs140570886 are on the same chromosome as the minor allele of rs186696265. This is in accordance with the observed SNP segregation patterns (Table S9 in the Supplemental Material). In the general population, these SNPs are in only partial linkage disequilibrium and have been independently linked to considerably increased Lp(a) concentrations (+43 to +64 mg/dL)^14,17^ and, in case of rs3798220, also increased OxPL load.^27^ All 3 SNPs were also significantly associated with increased *LPA* mRNA expression in liver in The Genotype-Tissue Expression Project (rs3798220: *P*=4.7×10^-8^; rs186696265: *P*=0.00073; rs140570886: *P*=1.2×10^-7^). Additionally, we observed at least one G allele of rs9347440 (minor allele frequency: 43.6% in Europeans; 59.4% in South Asians) in all hyper-Lp(a) individuals (Tables S8 and S9 in the Supplemental Material). This SNP has been previously linked to +250% in enhancer activity and +70% Lp(a) production.^28^ Its correlation with GWAS hits has not been reported. Other previously reported enhancer SNPs were inconclusive^28^ (Tables S8 and S9 in the Supplemental Material). No other variants segregating with the allele 21 were observed in a ≈5 kb around the enhancer regions DH-II and DH-III^5^ (except the frequent variant rs59872631, minor allele frequency=0.278) and in the >4 kb promoter region.^6,7^ Previously reported functional *LPA* SNPs are shown in Table S9 in the Supplemental Material. Of note, no role for rs10455872 was found, as this SNP was present only in two individuals carrying the 20 KIV allele that entered the pedigree by marriage (spouse II-C1 and his daughter III-C2; Table S9 in the Supplemental Material). Taken together, these results indicate that high-Lp(a) levels observed in this family are caused by a single high expressing *LPA* allele with 21 KIV characterized by the concomitant presence of multiple Lp(a)-increasing SNPs (rs3798220, rs186696265, rs140570886).

### Oxidized Phospholipids

*LPA* rs3798220 (Ile4399Met) has been associated with elevated oxPL in apoB containing lipoproteins.^27^ We thus measured the content of oxPL specifically associated with Lp(a) in all family members (Table S1 in the Supplemental Material). The levels of oxPL/Lp(a) were significantly higher in individuals carrying the 21 KIV isoform compared with the other family members (8.77±0.68 versus 7.45±1.65 nmol/L, respectively, *P*=0.01), indicating that the high expressing 21 KIV allele thus also carries an increased amount of OxPL onto Lp(a).

### GRS for Lp(a)

Principal component analysis using whole-genome microarray data indicated that most pedigree members clustered close to European, as well as Middle Eastern and South Asian groups (Figure S2 in the Supplemental Material), in line with the diverse and complex population history of La Réunion Island. In the present article, we, therefore, used the population-based European KORA F4 study (Kooperative Gesundheitsforschung in der Region Augsburg Follow Up Survey [F4]; n=3756) as primary reference population, as well as for validation the super populations Europeans (n=504) and South Asians, n=481) of the 1000 Genomes (1000G) project.

Because the SNPs mentioned above only explain a portion of the observed Lp(a) variance (see below), we hypothesized that multiple Lp(a)-increasing variants may sum up to create the observed high expressing phenotype in this family. We thus assessed the overall contribution of *LPA* variation to the observed phenotype using a 48-SNP GRS based on our recent GWAS on Lp(a),^17^ which captures the effects of 2001 genome-wide significant variants within the *LPA* locus. The Lp(a) GRS showed similar distribution in all 1000G super population and, most importantly, was centered at the same value (Figure S3 in the Supplemental Material).

**Figure 2.**
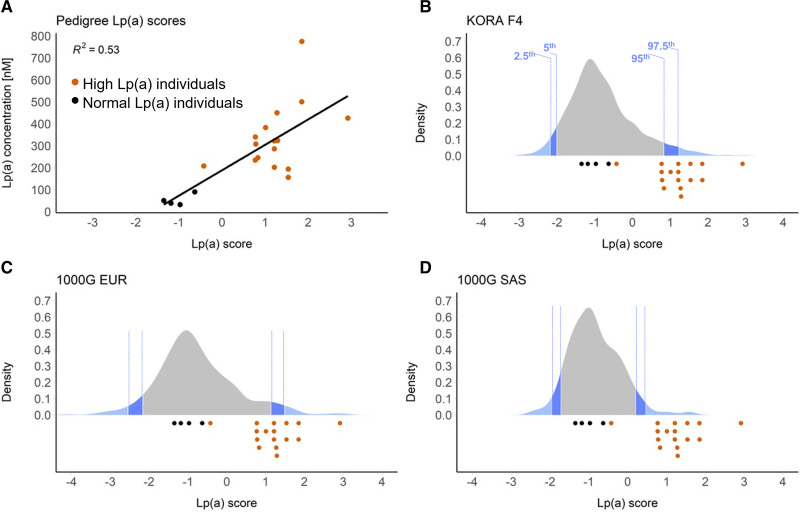
**Lp(a) (lipoprotein [a]) genetic risk scores. A**, Correlation between the Lp(a) Genetic risk scores (GRS) and Lp(a) plasma concentrations in family members with high (orange dots) and normal (black dots) Lp(a) levels. **B**, Distribution of *LPA* GRS in the general KORA F4 reference population. **C**, Distribution of Lp(a) GRS of the 1000 Genomes European (EUR) continental group. **D**, Distribution of Lp(a) GRS of the 1000 Genomes South Asian (SAS) continental group. Dark and light blue areas indicate bottom/top 5th and 2.5th percentiles, respectively. Lp(a) GRS of family members with high and normal Lp(a) levels (assessed in plasma samples three times in three independent experiments) are indicated by orange and black dots below each chart, respectively. For Lp(a) GRS, the effects of the 48 genome-wide significant single nucleotide polymorphisms (SNPs) were used. They represent 2001 genome-wide significant SNPs in a 1.76 Mb large region spanning *LPA* locus.

All pedigree members with high Lp(a) presented GRS values close to or above the 95th percentile of the GRS distribution of all 3 reference groups (Figure 2), except for individual III-C3, who carries only the isoform 15. In regression analysis, the GRS for Lp(a) explained 58.1% (*P*=9.341×10^-^^5^) of inverse-normal transformed Lp(a) concentrations. This was almost twice the variance explained by rs186696265 or rs140570886 alone (28.8%, *P*=0.0147). A model including the size of the shorter apo(a) isoform, age, sex, the presence of the KIV-2 splice site 4925G>A mutation,^15^ and the GRS for Lp(a) explained 75.9% of the Lp(a) variance (*P*=0.0006) in this family. This strongly argues for a cumulative impact of several Lp(a)-increasing variants that together create an overly high expressing isoform 21 allele.

### Impact on the GRS for CAD

**Figure 3.**
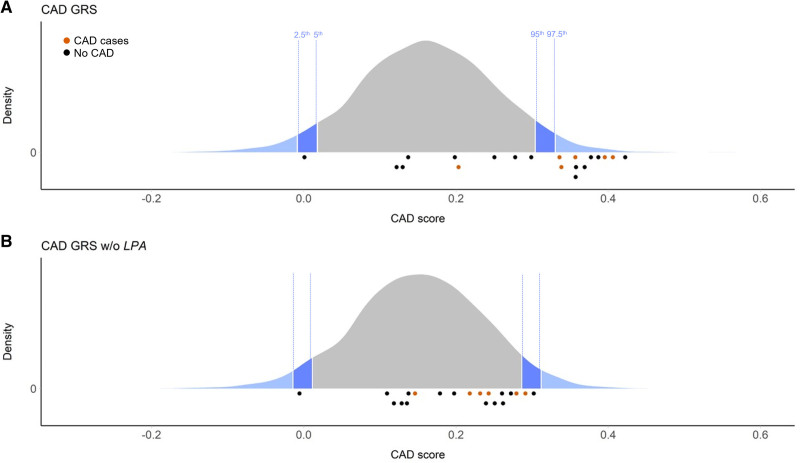
**Coronary artery disease (CAD) genetic risk scores. A**, Distribution of CAD genetic risk scores (GRS) in the general KORA F4 reference population. **B**, Distribution of CAD GRS in the general KORA F4 reference population when the *LPA* locus is removed from the score. Dark and light blue areas indicate bottom/top 5th and 2.5th percentiles, respectively. CAD GRS of family members who have and have not had CAD are indicated by orange and black dots below each chart, respectively. For CAD GRS, the effects of >≈6.6 million single nucleotide polymorphisms throughout the genome were used. The modified CAD GRS without the *LPA* locus was calculated by excluding all variants in the interval chr6:159 991 850–161 753 083.

Although most family members show increased Lp(a), not all have experienced premature CAD. To assess the polygenic CAD risk background and the impact of the *LPA* locus on it, we computed the polygenic CAD GRS recently published by Khera et al^29^ for the pedigree and all reference groups. The CAD scores showed very similar distribution in 1000G Europeans, 1000G South Asians, and KORA F4. All CAD cases but one (II-C1) showed a CAD score above the 97.5th percentile of KORA F4 (Figure 3). Similar observations were made using the 1000G Europeans and South Asians reference groups (Figure S4 in the Supplemental Material).

Most notably, despite the fact that the genome-wide GRS for CAD encompasses 6.6 M SNPs, removal of the broader sense *LPA* locus (defined as the ≈1.76 megabases region that showed genome-wide significant hits in Mack et al^17^) strongly reduced the genetic CAD risk in this family, bringing most of their CAD GRS below the 95th percentile of the respective distribution in KORA F4 (Figure 3).

Finally, we also hypothesized that a higher propensity to thrombotic events might enhance the effects of Lp(a) on CAD and used a GRS for venous thromboembolism (VTE) as a proxy for a putative thrombophilic genetic background in this family. No increased genetic risk for venous thromboembolism was seen in this family using the GRS of Klarin et al^30^ (Figure S5 in the Supplemental Material), even if some family members carry the prothrombin variant G20210A previously reported for the index patient^20^ (Table S10 in the Supplemental Material). Taken together, these results demonstrate the major contribution of the *LPA* locus to the elevated genetic risk of CAD in this family.

## Discussion

The Lp(a) trait is mostly controlled by the complex *LPA* gene locus,^17,18^ but the metabolic pathways governing Lp(a) plasma concentrations remain elusive.^1^ Neither biochemical nor large GWAS studies have conclusively identified a catabolic receptor nor other genes with a major impact on Lp(a) concentrations.^17,18,26^ Despite this nearly monogenic regulation, most Lp(a) epidemiology currently focuses on population studies whereas family studies using whole-genome approaches have not been pursued. Indeed, although *LPA* has been clearly established as the primary locus regulating Lp(a) concentrations in the population, it is unclear whether also other rare gene defects exist that may cause hyper-Lp(a). We, therefore, performed a comprehensive genetic characterization of a unique pedigree characterized by high Lp(a) and increased CAD incidence. The hyper-Lp(a) phenotype in the present pedigree was found to segregate with a strongly expressed *LPA* allele with 21 KIV (isoform 21) with no obvious contribution of coding variation in other genes (Supplemental Notes).

The isoform 21 that segregated with the phenotype was characterized by presence of three *LPA* SNPs rs186696265, rs140570886, and rs3798220. Rs186696265 is located close to both known enhancer regions upstream of *LPA*^5^ and is the SNP with the strongest effect in the GWAS of Mack et al^17^ (+64.7 mg/dL and +49.1 mg/dL in base and isoform-adjusted model; +47.6 mg/dL and +24.8 mg/dL in respective joint models with all other independent GWAS hits). Rs140570886, located in the intron of KIV-6, was the second strongest SNP in the same study after adjusting the GWAS for apo(a) isoforms measured by Western blot to detect SNPs that modify Lp(a) beyond the isoform effect (single SNP model: +43.3 mg/dL; joint model: +23.8 mg/dL). Both SNPs contributed independently to Lp(a) concentrations even if included in the same regression model,^17^ indicating an at least partially additive effect. The third SNP rs3798220 was associated with a high expressing apo(a) short isoform in an Austrian family.^31^ The observation in Arai et al^27^ and in the present work that this missense variant is associated with an increased oxPL load per Lp(a) particle is probably the mechanism by which this variant might contribute to increased atherogenicity. All 3 SNP have been reported by several GWAS studies on Lp(a), dyslipidemias, CAD risk, and related phenotypes.^32^ An overly strongly expressed 21 KIV allele of the *LPA* locus, carrying a high load of Lp(a)-increasing variants might thus suffice as primary cause for the hyper-Lp(a) phenotype in this family.

We used a GRS to additionally quantify the cumulative contribution of the genetic variability at the *LPA* locus (≈2000 SNPs captured via linkage disequilibrium) to the hyper-Lp(a) phenotype in this family. All but one individual with high Lp(a) showed an Lp(a) score close to or above the top 5th percentile of the reference populations. The hyper-Lp(a) individuals could thus be efficiently discriminated from their relatives with normal Lp(a) using an Lp(a) GRS. Interestingly, the only individual with high Lp(a) but a low Lp(a) GRS was an individual who had not inherited isoform 21, but isoform 15. Despite being shorter, isoform 15 was associated with somewhat lower Lp(a) than isoform 21, supporting the notion that genetic variants modify Lp(a) concentrations beyond what is determined by the sole size of the isoforms.^11,12,15^ Our observations are in line with recent reports from the UK Biobank, where the Lp(a) GRS resembled closely the directly measured Lp(a) values^33^ and offered comparable improvement in risk prediction as directly measured Lp(a).^34^ In datasets with genetic information but not directly measured Lp(a), the Lp(a) GRS might thus be a valid surrogate for Lp(a) plasma levels, as the effect of the GRS on cardiovascular risk appears fully mediated by its effect on Lp(a) concentrations.^34–37^

However, these studies were done in a large population-based study and it is unclear how well an Lp(a) GRS might be discriminative between closely related individuals. Our data shows that an Lp(a) GRS is discriminative even within families, capturing at least the most highly expressing alleles like the present 21 KIV isoform.

The pedigree was also characterized by a high incidence of CAD at relatively young age. Hypothesizing that the role of Lp(a) in determining the CAD risk may be further modified by an unfavorable polygenic background, we quantified the genome-wide polygenic contribution to CAD risk using a recently published genomic CAD GRS.^29^ All CAD cases but one showed CAD GRS above the 97.5th percentile of the score distribution in KORA F4. Most notably, however, exclusion of the *LPA* region from the score computation significantly lowered the CAD risk in these individuals, putting mostly all of them below the 95th percentile. Given that the *LPA* locus chiefly determines Lp(a) plasma levels, this observation indirectly establishes that Lp(a) concentrations are a driving factor for CAD in this family. Considering that the CAD GRS contains 6.6 M SNPs, this is noteworthy and underscores the large contribution of the *LPA* locus to the genetic risk in this pedigree. A similar observation in UK Biobank has been posted recently on medRxiv,^38^ supporting that in high-Lp(a) individuals the CAD GRS is indeed strongly determined by the *LPA* locus. Accordingly, an additive and even partially multiplicative effect of high Lp(a) and family history of CAD was recently reported.^39^ Conversely, we did not observe any increase in VTE GRS in this pedigree. This may appear surprising, given the assumed prothrombotic effects of Lp(a) but recent large Mendelian randomization studies about Lp(a) and VTE have also been negative.^40–42^ Only one study has reported an association between very high Lp(a) and VTE,^43^ but this effect might not be properly captured using a VTE GRS. Although Lp(a) might not play a substantial role in systemic thrombosis, local prothrombotic effects at the site of atherosclerotic lesions are conceivable.

GRS may be rapidly approaching applications in the clinics, and a high GRS for CAD found in any given person or family will lead to the question “Which gene loci are primarily driving this risk?” In individuals with very high–Lp(a) plasma concentrations, it will also be relevant to determine which other genetic factors are contributing to their CAD GRS. The sharp reduction in the CAD GRS after exclusion of the *LPA* gene region observed in this pedigree pinpoints the *LPA* locus as the major cause in this pedigree and provides a strong rationale to use Lp(a)-lowering therapies currently into development that specifically target *LPA* mRNA and thereby reduce Lp(a) plasma levels.^44^

### Limitations

We acknowledge that our investigation focused on a single yet large pedigree. Our approach can, however, be generalized in cohorts including either many pedigrees or a large number of unrelated hyper-Lp(a) individuals and matching controls. Sequencing studies in such cohorts have the potential to provide considerably larger datasets than single family–based studies. The present work may guide such endeavors. We also acknowledge that the selection of appropriate reference populations for genetic studies is inherently difficult, given the diverse ethnic background of La Réunion Island, with roots in Europe, Middle East, Africa (including Madagascar), East and South Asia. Nevertheless, European ancestry at least of the *LPA* locus is supported by the observation that rs3798220 segregated with a short apo(a) isoform, an association seen in Europeans but not in South Asians.^45^ This variant is not found at all in Africans.^45^ Likewise, rs140570886 and rs186696265 are 2.5× to 5× rarer in South Asians than in Europeans and absent in Africans. We thus consider that our reference populations were appropriate, even if more ethnically diverse reference groups would have been ideal. Finally, our study assumes a causal SNP that is segregating within the pedigree in a Mendelian fashion. We are aware that we would not have sufficient power to detect allelic heterogeneity, that is, different mutations at the same locus causing the same phenotype, albeit this seems a rather unlikely possibility in the present pedigree.

### Conclusions

Although some case reports about hyper-Lp(a) individuals and pedigrees have been published, none displays a thorough genetic characterization with whole-genome and whole-exome technologies. We here demonstrate that the Lp(a) GRS can successfully capture a hyper-Lp(a) phenotype also within pedigrees, despite the considerably higher relatedness they present compared with the general population. We also demonstrate in high-Lp(a) individuals that the CAD GRS can be strongly determined by the *LPA* locus. Although direct Lp(a) quantification is the preferred measure, in a future with individual genomic data being broadly available, routine determination of Lp(a) GRS may provide an actionable screening tool for cardiovascular risk prediction both in pedigrees and population.

## Article Information

### Sources of Funding

Dr Coassin is supported by the Austrian Science Fund (FWF) project P31458-B34 and the Research Grant 2018 of the Austrian Atherosclerosis Society. K. Chemello and Dr Blanchard received a scholarship from the European Union (European Regional Development Fund INTERREG V) and the Région Réunion (Saint-Denis, Réunion, France). Dr Nativel is a recipient of a post-doctoral fellowship from the Région Réunion. Dr Lambert is supported by the Agence Nationale de la Recherche (Paris, France) Program Grant CHOPIN (Cholesterol Personalized Innovation) ANR-16-RHUS-0007 and Project Grant KRINGLE2 ANR-20-CE14-0009 as well as by La Fondation De France (FDF-00096274). The KORA study was initiated and financed by the Helmholtz Zentrum München – German Research Center for Environmental Health, which is funded by the German Federal Ministry of Education and Research (BMBF) and by the State of Bavaria. These agencies had no role in the design and conduct of the study, in the collection, analysis, and interpretation of the data, and in the preparation, review, or approval of the article.

### Disclosures

Dr Lambert reports research grants and personal fees from Nyrada Ltd. These industries had no role in the design and conduct of the study, in the collection, analysis, and interpretation of the data, and in the preparation, review, or approval of the article. The other authors report no conflicts.

### Supplemental Materials

Supplemental Methods

Supplemental Results

Supplemental Notes

Tables S1–S10

Figures S1–S5

References 46–73

## Supplementary Material



## References

[1] Kronenberg F Utermann G. Lipoprotein(a): resurrected by genetics. J Intern Med. 2013;273:6–30. doi: 10.1111/j.1365-2796.2012.02592.x 22998429 10.1111/j.1365-2796.2012.02592.x

[2] Langsted A Nordestgaard BG Kamstrup PR. Elevated lipoprotein(a) and risk of ischemic stroke. J Am Coll Cardiol. 2019;74:54–66. doi: 10.1016/j.jacc.2019.03.524 31272552 10.1016/j.jacc.2019.03.524

[3] Boffa MB Koschinsky ML. Oxidized phospholipids as a unifying theory for lipoprotein(a) and cardiovascular disease. Nat Rev Cardiol. 2019;16:305–318. doi: 10.1038/s41569-018-0153-2 30675027 10.1038/s41569-018-0153-2

[4] Tsimikas S. A Test in context: lipoprotein(a): diagnosis, prognosis, controversies, and emerging therapies. J Am Coll Cardiol. 2017;69:692–711. doi: 10.1016/j.jacc.2016.11.042 28183512 10.1016/j.jacc.2016.11.042

[5] Wade DP Puckey LH Knight BL Acquati F Mihalich A Taramelli R. Characterization of multiple enhancer regions upstream of the apolipoprotein(a) gene. J Biol Chem. 1997;272:30387–30399. doi: 10.1074/jbc.272.48.30387 9374529 10.1074/jbc.272.48.30387

[6] Bopp S Köchl S Acquati F Magnaghi P Pethö-Schramm A Kraft HG Utermann G Müller HJ Taramelli R. Ten allelic apolipoprotein[a] 5’ flanking fragments exhibit comparable promoter activities in HepG2 cells. J Lipid Res. 1995;36:1721–1728. 7595093

[7] Chennamsetty I Claudel T Kostner KM Baghdasaryan A Kratky D Levak-Frank S Frank S Gonzalez FJ Trauner M Kostner GM. Farnesoid X receptor represses hepatic human APOA gene expression. J Clin Invest. 2011;121:3724–3734. doi: 10.1172/JCI45277 21804189 10.1172/JCI45277 PMC3163948

[8] Marcovina SM Hobbs HH Albers JJ. Relation between number of apolipoprotein(a) kringle 4 repeats and mobility of isoforms in agarose gel: basis for a standardized isoform nomenclature. Clin Chem. 1996;42:436–439. 8598109

[9] Boerwinkle E Leffert CC Lin J Lackner C Chiesa G Hobbs HH. Apolipoprotein(a) gene accounts for greater than 90% of the variation in plasma lipoprotein(a) concentrations. J Clin Invest. 1992;90:52–60. doi: 10.1172/JCI115855 1386087 10.1172/JCI115855 PMC443062

[10] Perombelon YF Soutar AK Knight BL. Variation in lipoprotein(a) concentration associated with different apolipoprotein(a) alleles. J Clin Invest. 1994;93:1481–1492. doi: 10.1172/JCI117126 8163653 10.1172/JCI117126 PMC294162

[11] Puckey LH Lawn RM Knight BL. Polymorphisms in the apolipoprotein(a) gene and their relationship to allele size and plasma lipoprotein(a) concentration. Hum Mol Genet. 1997;6:1099–1107. doi: 10.1093/hmg/6.7.1099 9215681 10.1093/hmg/6.7.1099

[12] Cohen JC Chiesa G Hobbs HH. Sequence polymorphisms in the apolipoprotein (a) gene. Evidence for dissociation between apolipoprotein(a) size and plasma lipoprotein(a) levels. J Clin Invest. 1993;91:1630–1636. doi: 10.1172/JCI116370 8473506 10.1172/JCI116370 PMC288140

[13] Kronenberg F. Prediction of cardiovascular risk by Lp(a) concentrations or genetic variants within the LPA gene region. Clin Res Cardiol Suppl. 2019;14(suppl 1):5–12. doi: 10.1007/s11789-019-00093-5 30859385 10.1007/s11789-019-00093-5

[14] Clarke R Peden JF Hopewell JC Kyriakou T Goel A Heath SC Parish S Barlera S Franzosi MG Rust S; PROCARDIS Consortium. Genetic variants associated with Lp(a) lipoprotein level and coronary disease. N Engl J Med. 2009;361:2518–2528. doi: 10.1056/NEJMoa0902604 20032323 10.1056/NEJMoa0902604

[15] Coassin S Erhart G Weissensteiner H Eca Guimarães de Araújo M Lamina C Schönherr S Forer L Haun M Losso JL Köttgen A. A novel but frequent variant in LPA KIV-2 is associated with a pronounced Lp(a) and cardiovascular risk reduction. Eur Heart J. 2017;38:1823–1831. doi: 10.1093/eurheartj/ehx174 28444229 10.1093/eurheartj/ehx174 PMC5837733

[16] Di Maio S Grüneis R Streiter G Lamina C Maglione M Schoenherr S Öfner D Thorand B Peters A Eckardt KU. Investigation of a nonsense mutation located in the complex KIV-2 copy number variation region of apolipoprotein(a) in 10,910 individuals. Genome Med. 2020;12:74. doi: 10.1186/s13073-020-00771-0 32825847 10.1186/s13073-020-00771-0 PMC7442989

[17] Mack S Coassin S Rueedi R Yousri NA Seppälä I Gieger C Schönherr S Forer L Erhart G Marques-Vidal P. A genome-wide association meta-analysis on lipoprotein (a) concentrations adjusted for apolipoprotein (a) isoforms. J Lipid Res. 2017;58:1834–1844. doi: 10.1194/jlr.M076232 28512139 10.1194/jlr.M076232 PMC5580897

[18] Hoekstra M Chen HY Rong J Dufresne L Yao J Guo X Tsai MY Tsimikas S Post WS Vasan RS. Genome-Wide Association Study Highlights APOH as a Novel Locus for Lipoprotein(a) Levels-Brief Report. Arterioscler Thromb Vasc Biol. 2021;41:458–464. doi: 10.1161/ATVBAHA.120.314965 33115273 10.1161/ATVBAHA.120.314965 PMC7769958

[19] Cameron SJ Block RC Richeson JF. Severe coronary disease in an adult considered at low cardiovascular disease risk with a healthy lifestyle. J Clin Lipidol. 2013;7:526–530. doi: 10.1016/j.jacl.2013.05.005 24079291 10.1016/j.jacl.2013.05.005

[20] Khantalin I Blanchard V Viallet N Lambert G. Recurrent coronary syndromes in a patient with isolated very-high lipoprotein (a) and the prothrombin genetic variant rs1799963 (G20210A): a case report. Eur Heart J Case Rep. 2019;3:ytz019. doi: 10.1093/ehjcr/ytz019 31020261 10.1093/ehjcr/ytz019 PMC6439380

[21] deGoma EM Wheeler MT Marcovina SM Ashley EA. Extremely elevated lipoprotein(a), combined hyperlipidemia, and premature atherosclerosis in a Chinese family. J Clin Lipidol. 2010;4:543–547. doi: 10.1016/j.jacl.2010.09.002 21122702 10.1016/j.jacl.2010.09.002

[22] Mobarek D Karasik PA Tomer M Miller M. High Lp(a) associated with very premature coronary heart disease. J Clin Lipidol. 2019;13:402–404. doi: 10.1016/j.jacl.2019.03.003 30987918 10.1016/j.jacl.2019.03.003

[23] Ellis KL Pang J Chan DC Hooper AJ Bell DA Burnett JR Watts GF. Familial combined hyperlipidemia and hyperlipoprotein(a) as phenotypic mimics of familial hypercholesterolemia: Frequencies, associations and predictions. J Clin Lipidol. 2016;10:1329–1337.e3. doi: 10.1016/j.jacl.2016.08.011 27919349 10.1016/j.jacl.2016.08.011

[24] Burgess S Ference BA Staley JR Freitag DF Mason AM Nielsen SF Willeit P Young R Surendran P Karthikeyan S; European Prospective Investigation Into Cancer and Nutrition–Cardiovascular Disease (EPIC-CVD) Consortium. Association of LPA variants with risk of coronary disease and the implications for lipoprotein(a)-lowering therapies: a mendelian randomization analysis. JAMA Cardiol. 2018;3:619–627. doi: 10.1001/jamacardio.2018.1470 29926099 10.1001/jamacardio.2018.1470 PMC6481553

[25] Said MA Yeung MW van de Vegte YJ Benjamins JW Dullaart RPF Ruotsalainen S Ripatti S Natarajan P Juarez-Orozco LE Verweij N. Genome-wide Association Study and identification of a protective missense variant on lipoprotein(a) concentration: protective missense variant on lipoprotein(a) concentration-brief report. Arterioscler Thromb Vasc Biol. 2021;41:1792–1800. doi: 10.1161/ATVBAHA.120.315300 33730874 10.1161/ATVBAHA.120.315300

[26] McCormick SPA Schneider WJ. Lipoprotein(a) catabolism: a case of multiple receptors. Pathology. 2019;51:155–164. doi: 10.1016/j.pathol.2018.11.003 30595508 10.1016/j.pathol.2018.11.003

[27] Arai K Luke MM Koschinsky ML Miller ER Pullinger CR Witztum JL Kane JP Tsimikas S. The I4399M variant of apolipoprotein(a) is associated with increased oxidized phospholipids on apolipoprotein B-100 particles. Atherosclerosis. 2010;209:498–503. doi: 10.1016/j.atherosclerosis.2009.09.077 19880117 10.1016/j.atherosclerosis.2009.09.077

[28] Puckey LH Knight BL. Sequence and functional changes in a putative enhancer region upstream of the apolipoprotein(a) gene. Atherosclerosis. 2003;166:119–127. doi: 10.1016/s0021-9150(02)00315-5 12482558 10.1016/s0021-9150(02)00315-5

[29] Khera AV Chaffin M Aragam KG Haas ME Roselli C Choi SH Natarajan P Lander ES Lubitz SA Ellinor PT. Genome-wide polygenic scores for common diseases identify individuals with risk equivalent to monogenic mutations. Nat Genet. 2018;50:1219–1224. doi: 10.1038/s41588-018-0183-z 30104762 10.1038/s41588-018-0183-z PMC6128408

[30] Klarin D Busenkell E Judy R Lynch J Levin M Haessler J Aragam K Chaffin M Haas M Lindström S; INVENT Consortium; Veterans Affairs’ Million Veteran Program. Genome-wide association analysis of venous thromboembolism identifies new risk loci and genetic overlap with arterial vascular disease. Nat Genet. 2019;51:1574–1579. doi: 10.1038/s41588-019-0519-3 31676865 10.1038/s41588-019-0519-3 PMC6858581

[31] Nikkola E Ko A Alvarez M Cantor RM Garske K Kim E Gee S Rodriguez A Muxel R Matikainen N. Family-specific aggregation of lipid GWAS variants confers the susceptibility to familial hypercholesterolemia in a large Austrian family. Atherosclerosis. 2017;264:58–66. doi: 10.1016/j.atherosclerosis.2017.07.024 28772107 10.1016/j.atherosclerosis.2017.07.024 PMC5698088

[32] Buniello A MacArthur JAL Cerezo M Harris LW Hayhurst J Malangone C McMahon A Morales J Mountjoy E Sollis E. The NHGRI-EBI GWAS Catalog of published genome-wide association studies, targeted arrays and summary statistics 2019. Nucleic Acids Res. 2019;47(D1):D1005–D1012. doi: 10.1093/nar/gky1120 30445434 10.1093/nar/gky1120 PMC6323933

[33] Dron JS Wang M Patel AP Kartoun U Ng K Hegele RA Khera AV. Genetic predictor to identify individuals with high lipoprotein(a) concentrations. Circ Genom Precis Med. 2021;14:e003182. doi: 10.1161/CIRCGEN.120.003182 33522245 10.1161/CIRCGEN.120.003182 PMC7887018

[34] Trinder M Uddin MM Finneran P Aragam KG Natarajan P. Clinical utility of lipoprotein(a) and LPA genetic risk score in risk prediction of incident atherosclerotic cardiovascular disease. JAMA Cardiol. 2020;6:1–9. 10.1001/jamacardio.2020.5398 PMC7539232 33021622

[35] Sandholzer C Saha N Kark JD Rees A Jaross W Dieplinger H Hoppichler F Boerwinkle E Utermann G. Apo(a) isoforms predict risk for coronary heart disease. A study in six populations. Arterioscler Thromb. 1992;12:1214–1226. doi: 10.1161/01.atv.12.10.1214 1390593 10.1161/01.atv.12.10.1214

[36] Gudbjartsson DF Thorgeirsson G Sulem P Helgadottir A Gylfason A Saemundsdottir J Bjornsson E Norddahl GL Jonasdottir A Jonasdottir A. Lipoprotein(a) Concentration and Risks of Cardiovascular Disease and Diabetes. J Am Coll Cardiol. 2019;74:2982–2994. doi: 10.1016/j.jacc.2019.10.019 31865966 10.1016/j.jacc.2019.10.019

[37] Schachtl-Riess JF Kheirkhah A Grüneis R Di Maio S Schoenherr S Streiter G Losso JL Paulweber B Eckardt KU Köttgen A; GCKD Investigators. Frequent LPA KIV-2 variants lower lipoprotein(a) concentrations and protect against coronary artery disease. J Am Coll Cardiol. 2021;78:437–449. doi: 10.1016/j.jacc.2021.05.037 34325833 10.1016/j.jacc.2021.05.037 PMC7613585

[38] Trinder M Brunham LR. Polygenic modulation of lipoprotein(a)-associated cardiovascular risk. medRxiv (Preprint). doi: 10.1101/2020.02.22.20026757

[39] Mehta A Virani SS Ayers CR Sun W Hoogeveen RC Rohatgi A Berry JD Joshi PH Ballantyne CM Khera A. Lipoprotein(a) and family history predict cardiovascular disease risk. J Am Coll Cardiol. 2020;76:781–793. doi: 10.1016/j.jacc.2020.06.040 32792075 10.1016/j.jacc.2020.06.040

[40] Larsson SC Gill D Mason AM Jiang T Bäck M Butterworth AS Burgess S. Lipoprotein(a) in Alzheimer, atherosclerotic, cerebrovascular, thrombotic, and valvular disease: mendelian randomization investigation. Circulation. 2020;141:1826–1828. doi: 10.1161/CIRCULATIONAHA.120.045826 32479194 10.1161/CIRCULATIONAHA.120.045826 PMC7614586

[41] Helgadottir A Gretarsdottir S Thorleifsson G Holm H Patel RS Gudnason T Jones GT van Rij AM Eapen DJ Baas AF. Apolipoprotein(a) genetic sequence variants associated with systemic atherosclerosis and coronary atherosclerotic burden but not with venous thromboembolism. J Am Coll Cardiol. 2012;60:722–729. doi: 10.1016/j.jacc.2012.01.078 22898070 10.1016/j.jacc.2012.01.078

[42] Kamstrup PR Tybjærg-Hansen A Nordestgaard BG. Genetic evidence that lipoprotein(a) associates with atherosclerotic stenosis rather than venous thrombosis. Arterioscler Thromb Vasc Biol. 2012;32:1732–1741. doi: 10.1161/ATVBAHA.112.248765 22516069 10.1161/ATVBAHA.112.248765

[43] Nordestgaard BG Langsted A. Lipoprotein (a) as a cause of cardiovascular disease: insights from epidemiology, genetics, and biology. J Lipid Res. 2016;57:1953–1975. doi: 10.1194/jlr.R071233 27677946 10.1194/jlr.R071233 PMC5087876

[44] Tsimikas S Karwatowska-Prokopczuk E Gouni-Berthold I Tardif JC Baum SJ Steinhagen-Thiessen E Shapiro MD Stroes ES Moriarty PM Nordestgaard BG; AKCEA-APO(a)-LRx Study Investigators. Lipoprotein(a) reduction in persons with cardiovascular disease. N Engl J Med. 2020;382:244–255. doi: 10.1056/NEJMoa1905239 31893580 10.1056/NEJMoa1905239

[45] Khalifa M Noureen A Ertelthalner K Bandegi AR Delport R Firdaus WJ Geethanjali FS Luthra K Makemaharn O Pang RW. Lack of association of rs3798220 with small apolipoprotein(a) isoforms and high lipoprotein(a) levels in East and Southeast Asians. Atherosclerosis. 2015;242:521–528. doi: 10.1016/j.atherosclerosis.2015.07.015 26302166 10.1016/j.atherosclerosis.2015.07.015

[46] Taliun D Harris DN Kessler MD Carlson J Szpiech ZA Torres R Taliun SAG Corvelo A Gogarten SM Kang HM; NHLBI Trans-Omics for Precision Medicine (TOPMed) Consortium. Sequencing of 53,831 diverse genomes from the NHLBI TOPMed Program. Nature. 2021;590:290–299. doi: 10.1038/s41586-021-03205-y 33568819 10.1038/s41586-021-03205-y PMC7875770

[47] Karczewski KJ Francioli LC Tiao G Cummings BB Alföldi J Wang Q Collins RL Laricchia KM Ganna A Birnbaum DP; Genome Aggregation Database Consortium. The mutational constraint spectrum quantified from variation in 141,456 humans. Nature. 2020;581:434–443. doi: 10.1038/s41586-020-2308-7 32461654 10.1038/s41586-020-2308-7 PMC7334197

[48] GTEx Consortium. The GTEx Consortium atlas of genetic regulatory effects across human tissues. Science. 2020;369:1318–1330. doi: 10.1126/science.aaz1776 32913098 10.1126/science.aaz1776 PMC7737656

[49] Viney NJ Yeang C Yang X Xia S Witztum JL Tsimikas S. Relationship between “LDL-C”, estimated true LDL-C, apolipoprotein B-100, and PCSK9 levels following lipoprotein(a) lowering with an antisense oligonucleotide. J Clin Lipidol. 2018;12:702–710. doi: 10.1016/j.jacl.2018.02.014 29574075 10.1016/j.jacl.2018.02.014

[50] Bertoia ML Pai JK Lee JH Taleb A Joosten MM Mittleman MA Yang X Witztum JL Rimm EB Tsimikas S. Oxidation-specific biomarkers and risk of peripheral artery disease. J Am Coll Cardiol. 2013;61:2169–2179. doi: 10.1016/j.jacc.2013.02.047 23541965 10.1016/j.jacc.2013.02.047 PMC3756816

[51] Taleb A Witztum JL Tsimikas S. Oxidized phospholipids on apoB-100-containing lipoproteins: a biomarker predicting cardiovascular disease and cardiovascular events. Biomark Med. 2011;5:673–694. doi: 10.2217/bmm.11.60 22003918 10.2217/bmm.11.60 PMC3230643

[52] Capoulade R Chan KL Yeang C Mathieu P Bossé Y Dumesnil JG Tam JW Teo KK Mahmut A Yang X. Oxidized phospholipids, lipoprotein(a), and progression of calcific aortic valve stenosis. J Am Coll Cardiol. 2015;66:1236–1246. doi: 10.1016/j.jacc.2015.07.020 26361154 10.1016/j.jacc.2015.07.020

[53] Blanchard V Ramin-Mangata S Billon-Crossouard S Aguesse A Durand M Chemello K Nativel B Flet L Chétiveaux M Jacobi D. Kinetics of plasma apolipoprotein E isoforms by LC-MS/MS: a pilot study. J Lipid Res. 2018;59:892–900. doi: 10.1194/jlr.P083576 29540575 10.1194/jlr.P083576 PMC5928431

[54] Blanchard V Garçon D Jaunet C Chemello K Billon-Crossouard S Aguesse A Garfa A Famchon G Torres A Le May C. A high-throughput mass spectrometry-based assay for large-scale profiling of circulating human apolipoproteins. J Lipid Res. 2020;61:1128–1139. doi: 10.1194/jlr.D120000835 32404332 10.1194/jlr.D120000835 PMC7328037

[55] Gries A Nimpf J Nimpf M Wurm H Kostner GM. Free and Apo B-associated Lpa-specific protein in human serum. Clin Chim Acta. 1987;164:93–100. doi: 10.1016/0009-8981(87)90110-0 2953508 10.1016/0009-8981(87)90110-0

[56] Chemello K Beeské S Trang Tran TT Blanchard V Villard EF Poirier B Le Bail JC Dargazanli G Ho-Van-Guimbal S Boulay D. Lipoprotein(a) cellular uptake ex vivo and hepatic capture in vivo is insensitive to PCSK9 inhibition with alirocumab. JACC Basic to Transl Sci. 2020;5:549–557. doi: 10.1016/j.jacbts.2020.03.008 10.1016/j.jacbts.2020.03.008 PMC7315184 32613143

[57] Erdel M Hubalek M Lingenhel A Kofler K Duba HC Utermann G. Counting the repetitive kringle-IV repeats in the gene encoding human apolipoprotein(a) by fibre-FISH. Nat Genet. 1999;21:357–358. doi: 10.1038/7681 10192381 10.1038/7681

[58] Li H Handsaker B Wysoker A Fennell T Ruan J Homer N Marth G Abecasis G Durbin R; 1000 Genome Project Data Processing Subgroup. The sequence alignment/map format and SAMtools. Bioinformatics. 2009;25:2078–2079. doi: 10.1093/bioinformatics/btp352 19505943 10.1093/bioinformatics/btp352 PMC2723002

[59] Danecek P Auton A Abecasis G Albers CA Banks E DePristo MA Handsaker RE Lunter G Marth GT Sherry ST; 1000 Genomes Project Analysis Group. The variant call format and VCFtools. Bioinformatics. 2011;27:2156–2158. doi: 10.1093/bioinformatics/btr330 21653522 10.1093/bioinformatics/btr330 PMC3137218

[60] Quinlan AR Hall IM. BEDTools: a flexible suite of utilities for comparing genomic features. Bioinformatics. 2010;26:841–842. doi: 10.1093/bioinformatics/btq033 20110278 10.1093/bioinformatics/btq033 PMC2832824

[61] Cingolani P Platts A Wang le L Coon M Nguyen T Wang L Land SJ Lu X Ruden DM. A program for annotating and predicting the effects of single nucleotide polymorphisms, SnpEff: SNPs in the genome of drosophila melanogaster strain w1118; iso-2; iso-3. Fly (Austin). 2012;6:80–92. doi: 10.4161/fly.19695 22728672 10.4161/fly.19695 PMC3679285

[62] Cingolani P Patel VM Coon M Nguyen T Land SJ Ruden DM Lu X. Using drosophila melanogaster as a model for genotoxic chemical mutational studies with a new program, SnpSift. Front Genet. 2012;3:35. doi: 10.3389/fgene.2012.00035 22435069 10.3389/fgene.2012.00035 PMC3304048

[63] McCarthy S Das S Kretzschmar W Delaneau O Wood AR Teumer A Kang HM Fuchsberger C Danecek P Sharp K; Haplotype Reference Consortium. A reference panel of 64,976 haplotypes for genotype imputation. Nat Genet. 2016;48:1279–1283. doi: 10.1038/ng.3643 27548312 10.1038/ng.3643 PMC5388176

[64] Loh PR Danecek P Palamara PF Fuchsberger C A Reshef Y K Finucane H Schoenherr S Forer L McCarthy S Abecasis GR. Reference-based phasing using the haplotype reference consortium panel. Nat Genet. 2016;48:1443–1448. doi: 10.1038/ng.3679 27694958 10.1038/ng.3679 PMC5096458

[65] Das S Forer L Schönherr S Sidore C Locke AE Kwong A Vrieze SI Chew EY Levy S McGue M. Next-generation genotype imputation service and methods. Nat Genet. 2016;48:1284–1287. doi: 10.1038/ng.3656 27571263 10.1038/ng.3656 PMC5157836

[66] Wichmann HE Gieger C Illig T; MONICA/KORA Study Group. KORA-gen–resource for population genetics, controls and a broad spectrum of disease phenotypes. Gesundheitswesen. 2005;67(Suppl 1):S26–S30. doi: 10.1055/s-2005-858226 16032514 10.1055/s-2005-858226

[67] Taliun D Chothani SP Schönherr S Forer L Boehnke M Abecasis GR Wang C. LASER server: ancestry tracing with genotypes or sequence reads. Bioinformatics. 2017;33:2056–2058. doi: 10.1093/bioinformatics/btx075 28200055 10.1093/bioinformatics/btx075 PMC5870850

[68] Li JZ Absher DM Tang H Southwick AM Casto AM Ramachandran S Cann HM Barsh GS Feldman M Cavalli-Sforza LL. Worldwide human relationships inferred from genome-wide patterns of variation. Science. 2008;319:1100–1104. doi: 10.1126/science.1153717 18292342 10.1126/science.1153717

[69] Elbitar S Susan-Resiga D Ghaleb Y El Khoury P Peloso G Stitziel N Rabès JP Carreau V Hamelin J Ben-Djoudi-Ouadda A. New sequencing technologies help revealing unexpected mutations in autosomal dominant hypercholesterolemia. Sci Rep. 2018;8:1943. doi: 10.1038/s41598-018-20281-9 29386597 10.1038/s41598-018-20281-9 PMC5792649

[70] Landrum MJ Lee JM Benson M Brown GR Chao C Chitipiralla S Gu B Hart J Hoffman D Jang W. ClinVar: improving access to variant interpretations and supporting evidence. Nucleic Acids Res. 2018;46(D1):D1062–D1067. doi: 10.1093/nar/gkx1153 29165669 10.1093/nar/gkx1153 PMC5753237

[71] Yang X Sethi A Yanek LR Knapper C Nordestgaard BG Tybjærg-Hansen A Becker DM Mathias RA Remaley AT Becker LC. SCARB1 gene variants are associated with the phenotype of combined high high-density lipoprotein cholesterol and high lipoprotein (a). Circ Cardiovasc Genet. 2016;9:408–418. doi: 10.1161/CIRCGENETICS.116.001402 27651445 10.1161/CIRCGENETICS.116.001402 PMC12199906

[72] Langsted A Nordestgaard BG Benn M Tybjærg-Hansen A Kamstrup PR. PCSK9 R46L loss-of-function mutation reduces lipoprotein(a), ldl cholesterol, and risk of aortic valve stenosis. J Clin Endocrinol Metab. 2016;101:3281–3287. doi: 10.1210/jc.2016-1206 27218270 10.1210/jc.2016-1206

[73] Lambert G Thedrez A Croyal M Ramin-Mangata S Couret D Diotel N Nobécourt-Dupuy E Krempf M LeBail JC Poirier B. The complexity of lipoprotein (a) lowering by PCSK9 monoclonal antibodies. Clin Sci (Lond). 2017;131:261–268. doi: 10.1042/CS20160403 28108631 10.1042/CS20160403

