# Supplementary material for: Genome-Wide Characterization of a Highly Penetrant Form of Hyperlipoprotein(a)emia Associated With Genetically Elevated Cardiovascular Risk
Source: Circ Genom Precis Med. 2022 Feb 7;15(2):e003489. doi: 10.1161/CIRCGEN.121.003489 (PMC9018215; doi:10.1161/CIRCGEN.121.003489)

SUPPLEMENTAL MATERIAL

SUPPLEMENTAL METHODS

Cascade screening and specimen collection - The index patient[20] and his adult relatives were invited to participate in a cascade screening project. Demographic parameters, medical history and clinical data were recorded. Blood samples were taken from the forearm of each participant following an overnight fast at first study visit. Plasma was isolated by centrifugation, aliquoted and stored at -80°C until use. Buffy coats were collected and DNA was isolated using the DNeasy Blood and Tissue kit (Qiagen, Courtaboeuf, France).

Genetic analyses - Whole exome sequencing, data processing and filtering, targeted sequencing and genotyping, whole genome genotyping and imputation against the haplotype reference consortium, genetic risk scores (GRS) calculations, description of reference populations and ancestry estimation, were performed according to protocols fully detailed in **Supplemental Methods** and **Supplemental Tables IV, V**. To assess whether the functional enhancer SNPs previously reported by Puckey et al[28] and the SNP rs186696265 (located close to the *LPA* enhancer regions[17]) and (rs9347440, rs7758766, rs7760010) were located on the gene allele with 21 KIV, the 21 KIV allele of one representative individual (II-A2) was isolated using an long-fragment pulsed-field gel electrophoresis protocol with Kpn2I digest [16,45]. Kpn2I excises a large fragment spanning over the whole *LPA* gene[16,45]. The two *LPA* gene alleles were isolated from the gel using the peqGOLD Gel Extraction Kit (VWR, Radnor, PA, US) and the target regions were amplified by PCR and sequenced using Sanger sequencing. The allelic location of rs186696265 was inferred to all individuals carrying the 21 KIV allele, as no sign of recombination was observed and the 21 KIV alleles in the pedigree were thus regarded as identical-by-descent. Phase of rs186696265, rs3798220 and rs140570886 was determined from the imputation.

Statistics - Statistics was done using R version 4.0.2. Amount of variance explained by the Lp(a) GRS was assessed by linear regression after stepwise adjustment for age and sex, isoform 1, and 4925 G>A[15] carrier status. *LPA* Arg21Ter[16] was determined in all individuals but was wild type in all. *APOE* status was not included in the regression as all samples showed the apoE phenotype ε3/ε3 except one ε2/ε3 and one ε3/ε4. The pedigree was drawn using the R package *kinship2*. Unless specified differently, minor allele frequencies (MAF) are from TOPMed freeze 8 [46] or from GnomAD Genomes [47]. The GTEx [48] v8 expression quantitative locus trait data for liver tissue was downloaded from the GTEx website (https://www.gtexportal.org/home/datasets). Expression quantitative locus trait data was available for 208 liver specimens, with 15, 12 and 10 specimens carrying minor alleles of rs3798220, rs140570886 and rs186696265, respectively. Plasma lipids, lipoproteins, and oxPL/Lp(a) are presented as mean ± SD and were compared between carriers and non-carriers of the 21KIV allele using Mann-Whitney test. Lp(a) GRS (explanatory variable) and inverse-normal transformed Lp(a) concentrations (dependent variable) were correlated using linear regression on R version 4.0.2. Both tests did not account for family relatedness, as all participants are from the same family with no additional cluster or levels of observation.

Plasma Lipids and Lipoproteins – Plasma total cholesterol (TC), triglycerides (TG) and high-density lipoprotein cholesterol (HDL-C) levels were measured using standard colorimetric assays in accredited laboratories. LDL-C levels were calculated using the Friedewald formula. Lp(a)-cholesterol [Lp(a)-C] was estimated by dividing Lp(a) particle numbers in nmol/L by 2.4 and then multiplying this by 0.3, which is the mean cholesterol content per unit of Lp(a) mass[49]. LDL-C levels were corrected by subtracting Lp(a)-C.

Quantification of oxidized phospholipids (oxPL) on Lp(a) – Anti-Apo(a) antibody-coated 8-well-strips (STA-359, Cell Biolabs, San-Diego CA, USA) were used to capture Lp(a) from human plasma samples diluted in PBS-0.1% casein, at final Lp(a) concentrations 100x above that necessary to saturate the capture antibody, for 2 h at 37°C. Saturation of the capture antibody was ascertained by measuring in parallel Lp(a) using the STA-359 detection antibody. Plates were subsequently washed 3 times with STA-359 wash buffer before each of the following steps: (i) The anti-oxPL biotinylated mouse E06 antibody (Avanti Polar Lipids, Alabaster AL, USA) diluted in STA-359 assay diluent at a 1µg/mL final concentration was added to each well for 1 hour at room temperature on an orbital shaker. (ii) The STA-359 Streptavidin-HRP conjugate was added to each well for 1 h at room temperature on an orbital shaker. (iii) The STA-359 Substrate Solution was added to each well for ~10 min and the reaction stopped by adding 100 µL of STA-359 Stop Solution. Absorbance was read at 450 nm on a spectrophotometer.
Serial dilutions of phosphocholine modified bovine serum albumin (PC-BSA) (Biosearch Technologies, Petaluma CA, USA) containing 2 moles of PC per mole of BSA, were plated in empty 8 well-strips to generate a standard curve, given that the E06 antibody similarly detects BSA bound phosphocholine and oxPL phosphocholine head groups [50,51]. These 8 well-strips were blocked with PBS-0.1% casein for 3 h at room temperature, prior to incubation and detection of the biotinylated mouse E06 antibody, as described above. The oxPL/Lp(a) values expressed in nM reflect the absolute content of oxPL for the same amount of captured Lp(a) and are therefore independent of plasma Lp(a) levels[52].

Apolipoproteins measurements — Plasma apolipoprotein (a), B100, and E molar concentrations as well as apoE phenotyping based on major isoforms (E2/E3/E4) were determined by liquid chromatography-high resolution mass spectrometry (LC-HRMS). In brief, plasma samples were prepared using a validated method involving trypsin proteolysis and subsequent analysis of proteotypic peptides described in detail previously [53,54]. LC-HRMS analyses of peptides were carried out on a Dionex Ultimate 3000 UHPLC System (Thermo Fisher Scientific, San Jose CA, USA) interfaced to Q Exactive Plus Orbitrap mass spectrometer (Thermo Fisher Scientific) with a heated electrospray ionization (HESI-II) probe in positive mode. The optimized LC-HRMS and parallel reaction monitoring (PRM) parameters are detailed in **Supplemental Table II and III**. Data acquisition was performed using the Xcalibur software (Version 4.3, Thermo Fisher Scientific). Quantitation of proteotypic peptides was performed using the Skyline software version 19.1 (MacCoss Laboratory, Seattle WA, USA) using calibration curves plotted from standard solutions and expressed in molar units, assuming that 1 mole of peptide is equivalent to one mole of protein. One mole of apo(a) was considered equivalent to one mole of Lp(a)[55]. We used an Lp(a) value of 125 nmol/L (≈50 mg/dL) to set

the limit above which individuals display high Lp(a) and below which individuals display normal Lp(a)[1].

LDLR expression, LDL and Lp(a) uptake in human primary lymphocytes – In a subset of individuals from the family, peripheral blood mononuclear cells (PBMCs) were isolated using Ficoll Paque Plus (Sigma-Aldrich, Saint Quentin-Fallavier, France) and subsequently stored frozen at -80°C in RPMI culture medium (Life Technologies, Saint Aubin, France) containing 70% fetal calf serum (FCS) and 10% dimethylsulfoxyde (DMSO) until use. PBMCs from one homozygous LDLR-negative familial hypercholesterolemic patient [56] were used as negative controls. Freshly thawed PBMCs were seeded in flat bottom 96-well plates (2.10^5cells per well) in RPMI containing 10 mM Hepes, 1 mM sodium pyruvate and 0.5% FCS for 2 h at 37°C. The culture medium was subsequently supplemented with 0 or 10 µg/mL mevastatin (Sigma) for 24h. Recombinant gain of function PCSK9-D_{374}Y (0 or 600 ng/mL) (Cyclex Co, Nagano, Japan) was added to the medium for the final 4h of the incubation time. In a subset of experiments, the PCSK9 inhibitor alirocumab was added concomitantly into the wells at a final concentration of 19.2 µg/mL.
For cell surface LDLR expression determination, PBMCs were washed twice in ice cold PBS containing 1% bovine serum albumin (PBS-1% BSA), and incubated with an allophycocyanin-conjugated antibody against the human LDLR (clone 472413) or an IgG1 (clone 11711) isotype control (R&D Systems, Lille, France) at 0.625 µg/mL for 20 min at room temperature in the dark. PBMCs were then washed twice in ice cold PBS-1% BSA and once in ice cold PBS. Cells were analyzed on a Cytoflex flow cytometer (Beckman Coulter, Indianapolis IN, USA). Forward scatter versus side-scatter gates were set to include only viable lymphocytes. A minimum of 5000 lymphocytes was analysed using the CytExpert software (Beckman Coulter). Mean fluorescence intensity (MFI) of cells incubated with the isotype control fluorescent antibody (non-specific binding) was subtracted from the MFI of cells incubated with a specific anti-LDLR fluorescent antibody to determine specific MFI levels [ΔMFI] of LDLR cell surface expression.
For fluorescent LDL and Lp(a) uptake assessments, LDL-bodipy or Lp(a)-bodipy [56] was added to the medium at a 10µg/mL final concentration for the final 3 h of the incubation time. PBMCs were washed twice in ice-cold PBS-1% BSA, once in ice-cold PBS and re-suspended in ice-cold PBS supplemented with 0.2% trypan blue (Sigma) to quench cell surface-bound fluorescent LDL or Lp(a) prior to flow cytometry analysis, exactly as above. MFI of lymphocytes incubated without fluorescent lipoproteins (autofluorescence) was subtracted from the MFI of cells incubated with fluorescent lipoproteins to determine the specific MFI levels [ΔMFI] of LDL and Lp(a) uptake in those cells, respectively. ΔMFI are expressed in arbitrary units (AU) throughout.

Determination of apo(a) isoform sizes on agarose gel electrophoresis – Plasma samples from each patient were diluted in deionized water at apo(a) concentration of 1.33 pmol/µL and subsequently mixed with 4X loading buffer (100mM Tris, 2 mM EDTA, 200 mM dithiothreitol, 4% SDS, 20% glycerol, 0.1% bromophenol blue). Molecular weight standards were prepared similarly from a mix of 5 plasma samples from individuals carrying apo(a) isoforms with sizes verified by pulse-field electrophoresis and Fiber-FISH [57]. Samples and standards preparations

were heated for 5 minutes at 95 °C and 10µL were loaded onto 3% agarose-TBE gels. Electrophoresis was run in TBE buffer containing 0.1% EDTA for 5 h at 65 mA. Proteins were subsequently transferred onto a nitrocellulose membrane in Tris-glycine buffer containing 0.1% SDS and 10% methanol. Membranes were blocked for 1 h in PBS containing 0.05% Tween 20 and 5% fat-free milk powder. Membranes were then incubated for 1 h with the TA310972 anti-apo(a) rabbit monoclonal antibody (Origene, Rockville MD, USA) diluted 1/20,000 in blocking buffer. Membranes were washed three times 5 min in PBS containing 0.05% Tween and subsequently incubated for 1h with an HRP conjugated anti-rabbit IgG secondary antibody (Jackson ImmunoResearch, Ely, UK) diluted 1/10,000 in PBS containing 0.05% Tween and 5% BSA. Membranes were washed and bands were visualized by chemiluminescence using SuperSignal Pico PLUS (Thermo Fisher Scientific) on the Amersham Imager 680. Determination of the relative intensities of each apo(a) isoform was assessed by densitometric analysis using the Amersham Imager 680 imaging software (GE Healthcare Life Sciences, Velizy-Villacoublay, France). Throughout, the shorter apo(a) isoform visible by Western blot was termed isoform-1 and the longer isoform-2. When only one apo(a) isoform was detected it was considered isoform-1.

***Whole exome sequencing* —** Whole exome sequencing (WES) was performed on 13 out of 22 family members. The other individuals of the family enrolled after completion of these analyses (**Supplemental Table I**) but were mostly included in genome-wide microarray genotyping (see below). Sequencing was to >100X depth (12 Gb) at BGI (BGI Tech Solutions, Shenzen, China) using the Agilent V6 exome library kit (Agilent Technologies, Santa Clara, CA, USA) on an Illumina (Illumina, San Diego, CA, USA) HiSeq System using Paired-End 150-bp sequencing chemistry. Raw data processing was carried out at BGI Tech Solutions following the GATK best practices pipeline. All data is aligned to human genome reference GRCh37/hg19.

***Exome data processing and filtering* —** Data processing was done using samtools 1.9 [58], bcftools 1.9 [58], vcftools 0.1.17 [59] and bedtools 2.28.0 [60]. Annotation and case-control filtering was done using snpEff 4.3 [61] and snpSift 4.3 [62]. The analysis dataset was restricted to variants with ≥4-fold coverage located ±50 bp within annotated exons according to the UCSC genome browser hg19 NCBI RefSeq Release 105.20190906 (2019-10-24) and functionally affecting exonic sequences (variants classified as missense, located in 5' or 3' UTRs, creating of deleting stop or start codons or affecting splice acceptors or donors). An autosomal dominant mode of inheritance was assumed. Mendelian violations were assessed for quality control using Picard tools. Candidate genes inspection included all genes recently described as potential modulators of Lp(a) metabolism [26] (*ANXA2, LGALS1, TLR2, APOE, ASGR1, PLGRKT, TLR6, CD36, SCARB1, LRP1, LDLR, VLDLR, LRP2*), as well as *APOB* and *PCSK9.*

***Targeted sequencing and genotyping* —** For all family members but two for whom DNA was not collected (**Supplemental Table I**), two 5.2 kb, respectively 4.3 kb large regions containing the enhancer regions DHII and DHIII [5] and previously described *LPA* promoter regions [6,7] were amplified by PCR on genomic DNA and sequenced on an ABI 3130xl System (Thermo Fisher Scientific) (primer list and PCR conditions shown in **Supplemental Tables IV and V**). The genotypes of the GWAS top hit *LPA* rs186696265 [17], the prothrombin mutation

rs1799963/G20210A and the rare *APOB* variant rs781243278 (Arg2522Gln) found in the WES phase were determined (respectively confirmed) by Sanger sequencing. The genotypes of *LPA* rs140570886 [17], *LPA* rs10455872 and *LPA* rs3798220 [14] were retrieved from the imputed microarray data (rs140570886; imputation quality R^2: 0.966), genotyped directly on the microarray (rs10455872) or determined in the WES data (rs3798220). The *LPA* KIV-2 splice site mutation 4925G>A and *LPA* KIV-2 Arg21Ter stop mutation were assessed by allele-specific PCR as published previously [15,16].

Whole genome genotyping and imputation **-** All adult participants with DNA available were subjected to whole genome genotyping using the Global Screening Array (GSA) BeadChip (Illumina, San Diego CA, USA). This bead array was specifically designed to allow high imputation accuracy at minor allele frequencies >1% across all 26 populations of the 1000 Genomes (1000G) Project. After applying data quality and data cleaning steps (SNP and sample call rates equal or above 90%), 609,447 variants were used for genotype imputation. Microarray data was then imputed against the HRC reference panel [63,64] using the Michigan Imputation Server [65]. After imputation, 39,127,678 variants were used for all subsequent analyses.

The KORA F4 study **-** The KORA F4 study [66], conducted from 2006 to 2008, is a population-based sample from the general population living in the region of Augsburg, Southern Germany, which has evolved from the WHO MONICA study (Monitoring of Trends and Determinants of Cardiovascular Disease). The Affymetrix axiom array was used for genotyping. Individuals with Non-European ancestry and population outliers were excluded. Genotypes were imputed using the HRC reference panel.

Genetic risk score calculations and ancestry estimation **-–** The imputed genotypes of all family members with microarray data were used to calculate genetic risk scores (GRS) for Lp(a) (48 SNPs) and for coronary artery disease (CAD) (6.6 million variants).
For the Lp(a) GRS, the effects of the 48 genome-wide significant SNPs in regression model 1 reported in [17] were used. These represent 2,001 genome-wide significant SNPs in a 1.76 Mb large region spanning the *LPA* locus. The CAD GRS was derived from [29]. The modified CAD GRS without the *LPA* locus was calculated by excluding all variants in the interval chr6:159,991,850-161,753,083 (corresponds to the region that showed genome-wide significant hits in [17], i.e. the broader sense *LPA* locus).
Each imputed genotype dosage was multiplied by the corresponding weights and all values were summed up to the individual score. This score indicates the individual's likelihood of displaying the given phenotype. The same scores were calculated for KORA F4 (3,756 samples imputed against HRC) and the 1000 Genomes Project (2,504 samples). All scores were calculated using PGS-Calc (https://github.com/lukfor/pgs-calc).
The genetic background of the pedigree members was assessed by principal component analysis (PCA) using the LASER server [67] with the Human Genetic Diversity Panel (HGDP) as ancestry reference panel [68].

SUPPLEMENTAL RESULTS

Lp(a) cellular uptake – To ascertain that elevated Lp(a) plasma levels in this family do not result from any potential receptor defect, PBMCs were isolated from a subset of normolipoprotein(a)emic and hyperlipoprotein(a)emic family members as well as from one unrelated homozygous FH (HoFH) patient totally lacking the LDLR used as control. Cells were incubated sequentially with mevastatin, rPCSK9, and alirocumab to modulate the abundance of the LDLR at their surface (**Supplemental Figure I**). LDLR expression was similar at the surface of lymphocytes from normo- and hyper-lipoprotein(a)emic individuals, but as anticipated extremely low at the surface of HoFH lymphocytes (**Supplemental Figure IA**). Mevastatin increased whereas rPCSK9 reduced LDLR cell surface expression in lymphocytes from normo- and hyper-lipoprotein(a)emic individuals to similar extents. Alirocumab restored LDLR cell surface expression in lymphocytes from normo- and hyper-lipoprotein(a)emic individuals. Neither mevastatin nor rPCSK9 nor Alirocumab significantly modulated LDLR cell surface expression in HoFH lymphocytes. (**Supplemental Figure IA**).
Paralleling the levels of LDLR cell surface expression, LDL uptake was similar in lymphocytes from normo- and hyper-lipoprotein(a)emic individuals but minimal in HoFH lymphocytes. Likewise, mevastatin increased, rPCSK9 reduced, and Alirocumab restored LDL uptake to similar extents in lymphocytes from normo- and hyper-lipoprotein(a)emic individuals. Neither mevastatin nor rPCSK9 nor Alirocumab significantly altered LDL uptake in HoFH lymphocytes (**Supplemental Figure IB**). In sharp contrast with LDL uptake, Lp(a) cellular uptake was similar in HoFH lymphocytes and in lymphocytes isolated from normo- and hyper-lipoprotein(a)emic individuals. Neither mevastatin nor rPCSK9 nor alirocumab treatments significantly altered Lp(a) cellular uptake in any of the lymphocytes tested (**Supplemental Figure IC**). These results demonstrate that Lp(a) cellular uptake is unaffected in persons with hyper-lipoprotein(a)emia from this family and not differentially modulated by genetic or pharmacological modulations of LDLR function *ex-vivo*.

SUPPLEMENTAL NOTES

1- The rare *APOB* Arg2522Gln variant (rs781243278, MAF=3.4E-05) was found in 8 of 9 hyper-Lp(a) individuals but has no known effect on apoB containing lipoproteins plasma concentrations [69]. Also ClinVar [70] classifies Arg2522Gln as variant of unknown significance (https://www.ncbi.nlm.nih.gov/clinvar/RCV000422131/).

2- The *SCARB1* variant Gly2Ser (rs4238001, MAF=0.057-0.116, **Supplemental Table VII**) has been proposed to cause a combined high HDL-C and high Lp(a) phenotype before [71]. However, its role in our pedigree is unclear since **(i)** it was observed only in 7 of 9 hyper-Lp(a) individuals, **(ii)** their HDL-C levels were normal (all between 1.09 and 1.60 mmol/L) and **(iii)** the study by Yang et al [71] defined high Lp(a) using different cut-off values in the two populations studied. Moreover, despite *SCARB1* Gly2Ser being a frequent polymorphism, it did not reach genome-wide significance in a recent large GWAS for Lp(a) with ≈300,000 individuals [18]. As the latter found effect sizes in the range of 1 nmol/L for Lp(a), a major role for *SCARB1* Gyl2Ser appears unlikely.

3- A 3'UTR variant in *GM2A* and a missense variant in *SYNJ2* were sufficiently rare in Europeans or South Asians, the two ethnic groups most closely related to our family, to be plausible candidates but these genes present no biological connection to Lp(a) metabolism. *SYNJ2* (Synaptojanin 2) is an inositol polyphosphate 5-phosphatase that inhibits clathrin-mediated endocytosis. While its involvement in endocytosis would make it an appealing candidate, it is mainly expressed in neuronal tissue with only very minor expression in kidney and liver (according to GTEx [48]), which are the most likely catabolic sites of Lp(a). Moreover, it is located close to the *LPA* locus, but outside of the GWAS peaks for Lp(a) [17,18]. This makes it most likely a chance finding located on the same haplotype as rs3798200. *GM2A* (GM2 ganglioside activator) catalyzes the degradation of the ganglioside GM2 and loss-of-function mutations in *GM2A* cause GM2-gangliosidosis AB variant, a rare neuronal degeneration disease.

SUPPLEMENTAL TABLES

Supplemental Table I. Demographic, medical and biochemical information of each individual family member.

id	sex	MI (age)	YOB	Micro Array	WES	Rx	Current or past smoking	BMI (kg/m2)	HBP	T2D	TC (mmol/L)	TG (mmol/L)	HDL-C (mmol/L)	LDL-C (mmol/L)	Lp(a) (nmol/L)	Lp(a)-C (mmol/L)	LDL-C corr (mmol/L)	apoA1 (mg/dL)	apoB100 (mg/dL)	ApoE genotype	OxPL/Lp(a) (nM)
II-A1	M		1959	Y	Y			30.9			5.20	1.30	1.05	3.55	39	0.12	3.43	163	86	E3:E3	7.6
III-A4	F		1979	Y	Y			21.2			4.20	0.76	1.35	2.49	90	0.29	2.20	41	42	E3:E3	7.4
III-A1	F		1978	Y	Y			28.7	Y		4.80	0.70	1.57	2.90	340	1.09	1.80	186	83	E3:E3	8.1
III-A2	M		1980	Y	Y			22.3			4.00	1.00	1.09	2.50	308	0.99	1.50	144	89	E3:E3	8.0
III-A3	M	32	1981	Y	Y	LLT + Aph		22.5			3.49	0.62	1.33	1.88	235	0.75	1.12	138	76	E3:E3	8.2
II-C2	F		1958	Y	Y			26.9		Y	5.88	1.37	1.25	4.01	286	0.92	3.08	142	124	E3:E3	9.8
II-B1	M	52	1959	Y	Y	LLT	Y	21.4			4.00	0.80	1.13	2.50	383	1.23	1.26	132	99	E3:E3	9.7
II-A2	F		1961	Y	Y			32.9		Y	5.90	1.30	1.47	3.80	202	0.65	3.14	179	118	E3:E3	9.0
II-D1	F	50	1963	Y	Y	LLT	Y	29.4			3.60	1.00	1.09	2.10	322	1.04	1.06	144	75	E3:E3	9.3
III-A5	M		1983	Y	Y			31.4			5.40	1.30	1.60	3.20	324	1.04	2.15	213	119	E3:E3	8.5
II-B2	F		1953	Y	Y			31.3	Y		6.70	1.90	1.39	4.40	51	0.16	4.23	156	145	E3:E3	5.2
III-B1	M	31	1980	Y	Y	LLT		29.3	Y		3.30	0.50	1.34	1.70	246	0.79	0.90	136	65	E3:E3	9.0
III-B3	F		1982	Y	Y		Y	26.7			4.60	0.90	1.39	2.80	32	0.10	2.69	157	58	E3:E3	6.8
III-B4	M		1984	Y				22.5			5.06	0.66	1.37	3.38	194	0.62	2.75	143	77	E3:E3	8.7
III-B5	F		1991	Y				31.2			5.60	0.76	1.38	3.86	156	0.50	3.35	161	103	E3:E3	8.4
II-C1	M	38	1955	Y		LLT		26.4		Y	5.79	2.86	1.04	3.45	450	1.45	1.99	191	119	E2:E3	10.3
III-C1	F		1979	Y				21.2			5.45	1.76	1.14	3.43	500	1.61	1.81	265	121	E3:E3	7.6
III-C2	F		1982	Y				18.0			4.32	1.25	1.05	2.70	426	1.37	1.32	112	88	E3:E3	9.0
III-C3	F		1986	Y				23.7			6.58	1.31	1.96	4.02	208	0.67	3.34	208	100	E3:E3	7.4
III-C4	M	27	1991	Y		LLT	Y	24.1			6.52	2.20	1.23	4.31	775	2.50	1.80	157	84	E3:E3	8.4
II-D2	M		1966					23.4			4.42	1.00	2.25	1.72	360	1.16	0.55	131	78	E3:E3	9.8
III-A6	M		1979					23.5	Y		4.24	0.96	2.02	1.74	5	0.01	1.72	131	61	E3:E4	5.2

MI myocardial infarction, YOB year of birth, WES whole exome sequencing, Rx prescription, LLT lipid lowering treatment, Aph apheresis, BMI body mass index, HBP high blood pressure, T2D type 2 diabetes, LDL-C_{corr} LDL-C corrected for Lp(a) cholesterol, Lp(a)-C Lp(a) cholesterol, OxPL oxidized phospholipids, Y yes. Family members of the 2nd and 3rd generations are highlighted in purple and light blue colors respectively. All measurements have been performed three times independently.

Supplemental Table II. LC-HRMS parameters

**Column**	Aeris™ 1.7 µm, 100 × 2.1 mm, PEPTIDE XB-C18
**Mobile phase A**	0.1% formic acid in 5:95 acetonitrile:water (v/v)
**Mobile phase B**	0.1% formic acid in acetonitrile
**Linear gradient**	• B = 1% for 1 min<br>• 1% to 29% in 9 min<br>• 29% to 95% in 0.5 min<br>• 95% for 1 min<br>• 95% to 1% in 0.5 min<br>• 1% for 1.5 min
**Flow rate**	350 µL/min
**Injection volume**	30 µL
**Column temperature**	60 °C
**Polarity**	Positive HESI mode
**Capillary voltage**	3 kV
**Sheath gas flow rate**	48.75
**Aux gas flow rate**	11.88
**Capillary temperature**	250 °C
**Desolvation temperature**	400 °C
**Desolvation gas**	N_2
**HCD cell gas**	N_2
**Acquisition mode**	PRM (MS/MS)
**PRM transitions**	*See Supplemental table 3*
**Normalized Collision Energy (NCE)**	*See Supplemental table 3*
**Resolution**	35,000
**AGC target**	2e5
**Maximum IT**	120 ms
**Isolation window**	1.4 *m/z*

Supplemental Table III. Parallel reaction monitoring (PRM) parameters for proteotypic peptide detection (LC-HRMS).

**Apo**	**Sequence**	**Transitions (*m/z*)**	**NCE**	**Fragments**
ApoB100	ATGVLYDYVNK	621.8219 → 1070.5517, 914.4618, 801.3777, 523.2875	28	y_9^+ y_7^+ y_6^+ y_4^+
ApoB100 (IS)	ATGVLYDYVN-[$^{13}C_6$,$^{15}N_2$]K	625.8290 → 1078.5659, 922.4760, 809.3919, 531.3017		
Apo(a)	LFLEPTQADIALLK	786.4558 → 1069.6252, 743.4662, 557.4021, 444,3180	28	y_{10}^+ y_7^+ y_5^+ y_4^+
Apo(a) (IS)	LFLEPTQADIALL-[$^{13}C_6$,$^{15}N_2$]K	790.4629 → 1077.6394, 751.4804, 565.4163, 452.3322		
ApoE	LGPLVEQGR	484.7798 → 701.3941, 588.3100, 489.2416, 360.1990	28	y_6^+ y_5^+ y_4^+ y_3^+
ApoE (IS)	LGPLVEQG-[$^{13}C_6$,$^{15}N_4$]R	489.7840 → 711.4023, 598.3183, 499.2499, 370.2073		
ApoE2	(**C**)LAVYQAGAR	554.7820 → 835.4421, 764.4050, 665.3365, 502.2732	28	y_8^+ y_7^+ y_6^+ y_5^+
ApoE2 (IS)	(**C**)LAVYQAGA-[$^{13}C_6$,$^{15}N_4$]R	559.7862 → 845.4503, 774.4132, 675.3448, 512.2815		
ApoE4	LGADMEDVR	503.2371 → 892.3829, 835.3614, 764.3243, 649.2974	28	y_8^+ y_7^+ y_6^+ y_5^+
ApoE4 (IS)	LGADMEDV-[$^{13}C_6$,$^{15}N_4$]R	508.2413 → 902.3912, 845.3697, 774.3326, 659.3057		
ApoE2/E3	LGADMEV(**C**)GR	611.7632 → 981.3764, 866.3495, 735.3090, 491.2395	28	y_8^+ y_7^+ y_6^+ y_4^+
ApoE2/3 (IS)	LGADMEV(**C**)G-[$^{13}C_6$, $^{15}N_4$]R	616.7673 → 991.3847, 876/3578, 745.3173, 501.2477		
ApoE3/E4	LAVYQAGAR	474.7667 → 764.4050, 665.3365, 502.2732, 374.2146	28	y_7^+ y_6^+ y_5^+ y_4^+
ApoE3/E4 (IS)	LAVYQAGA-[$^{13}C_6$, $^{15}N_4$]R	479.7708 → 774.4132, 675.3448, 512.2815, 384.2229		

NCE: Normalized Collision Energy; (**C**): alkylated cysteine with a mass shift of +57.0214.

Supplemental Table IV. PCR conditions.

Target	Enhancer DHII	Enhancer DHIII	rs186696265	Promoter	Prothrombin rs1799963	ApoB rs781243278
**Product size [bp]**	3130	2055	391	4309	467	465
**Chromosome**	6	6	6	6	11	2
**Genome position (hg19, chr6)**	161113496-161116625	161104839-161106893	161111547-161111937	161085110-161089418	46760851-46761317	21231960-21232424
**Enzyme**	PfuUltra II Fusion HS DNA Polymerase	PfuUltra II Fusion HS DNA Polymerase	HotStarTaq DNA, Qiagen	KAPAHiFi HotStart DNA Polymerase, KAPA Biosystems	HotStarTaq DNA, Qiagen	Herculase II Fusion, Agilent
**Forward primer**	LPA_Enhancer_fw4	LPA_Enhancer_rv3	LPA_rs186696265_fw1	LPA_Prom_Chen_fw	rs1799963_fw1	APOB_Ex26_fw1
**Reverse primer**	LPA_Enhancer_rv5	LPA_Enhancer_fw5	LPA_rs186696265_rv1	Bopp.Tarmelli_Rv	rs1799963_rv1	APOB_Ex26_rv1
**Reaction volume [µL]**	10	10	10	10	10	10
**Enzyme amount [µL]**	0.2	0.2	0.05	0.2	0.5	0.1
**Primer, final concentration [µM each]**	0.2	0.2	0.2	0.2	0.2	0.25

**dNTP, final concentration [mM]**	0.1	0.1	0.2	0.3	0.2	0.25
**DNA input [ng]**	2	2	12.5	6.48	12.5	50
**Initial denaturation**	95°C, 2 min	95°C, 2 min	95°C, 15 min	95°C, 3 min	95°C, 15 min	95°C, 2 min
**Denaturation**	95°C, 20 sec	95°C, 20 sec	94°C, 30 sec	98°C, 20 sec	94°C, 30 sec	95°C, 20 sec
**Annealing**	63°C, 20 sec	58°C, 20 sec	58°C, 30 sec	65°C -0.5°C/cylce, 15 sec (5 cycles) 60°C, 15 sec (30 cycles)	54°C, 30 sec	57.6°C, 20 sec
**Extension**	72°C, 35 sec	72°C, 35 sec	72°C, 1 min	72°C, 6 min	72°C, 1 min	72°C, 30 sec
**Final extension**	72°C, 3 min	72°C, 3 min	72°C, 10 min	72°C, 4.5 min	72°C, 10 min	72°C, 3 min
**No. of cycles**	30	30	35	5+30	35	30

Supplemental Table V. PCR and sequencing primers

Use	PCR fragment *	PrimerID	Sequence 5' – 3'
PCR Primer	Enhancer DHII	LPA_Enhancer_fw4	ATGGTCCAATTGTGCAGGCT
PCR Primer	Enhancer DHII	LPA_Enhancer_rv5	GCATGCTTTGTGGAAGAAGGATTG
PCR Primer	Enhancer DHIII	LPA_Enhancer_rv3	TCTTCCTAGTCTCAATGGTCTTTAC
PCR Primer	Enhancer DHIII	LPA_Enhancer_fw5	TGGCTGGGAAAACCTATTGCT
PCR Primer	LPA_rs186696265	LPA_rs186696265_fw1	ACAAAGGAGTTGGGCAGC
PCR Primer	LPA_rs186696265	LPA_rs186696265_rv1	GTTCAGCCCAGGACAGC
PCR Primer	Promoter	LPA_Prom_Chen_fw	CAATAATGACATCAGGTCAATTGGC
PCR Primer	Promoter	Bopp.Taramelli_Rv	GAATTGCACATAAAGCCATGGC
PCR Primer	Prothrombin rs1799963	rs1799963_fw1	GTCTCATGGGGTGAAGGC
PCR Primer	Prothrombin rs1799963	rs1799963_rv1	CTCTCACCTGGCCCTGC
PCR Primer	ApoB rs781243278	APOB_Ex26_fw1	GGATGGTCTTGATTTCAGGAACAGTG
PCR Primer	ApoB rs781243278	APOB_Ex26_rv1	CCAATGACAAAATCCGTGAGGTG
Seq. Primer	Enhancer DHII	LPA_Enhancer_fw4	ATGGTCCAATTGTGCAGGCT
Seq. Primer	Enhancer DHII	LPA_Enhancer_fw11	GGGAATGAAATCAAGTATGCTGC
Seq. Primer	Enhancer DHII	LPA_Enhancer_rv14	TGTTGAGGACTTTATCCTAGGGC
Seq. Primer	Enhancer DHII	LPA_Enhancer_fw12	GCACTACCTTAGCAGAGTTGAGC
Seq. Primer	Enhancer DHII	LPA_Enhancer_fw6	CAACAACATAAAGACCCAGAAGGAG
Seq. Primer	Enhancer DHII	LPA_Enhancer_fw15	GCCTGTGTATGCTTTGATTGC

Seq. Primer	Enhancer DHII	LPA_Enhancer_rv8	CACAGGAACAGAAAACCAAATATCGC
Seq. Primer	Enhancer DHII	LPA_Enhancer_fw16	TCATTAGTTATTTTTCCTGACGC
Seq. Primer	Enhancer DHII	LPA_Enhancer_fw17	CATTACACCTCTAGGTAAGTCAGC
Seq. Primer	Enhancer DHII	LPA_Enhancer_rv10	ATGTACTCTTCTTACAGGATGAGGCC
Seq. Primer	Enhancer DHII	LPA_Enhancer_fw18	TCTCAAAGCCTTCCTCACGC
Seq. Primer	Enhancer DHII	LPA_Enhancer_rv5	GCATGCTTTGTGGAAGAAGGATTG
Seq. Primer	Enhancer DHIII	LPA_Enhancer_fw5	TGGCTGGGAAAACCTATTGCT
Seq. Primer	Enhancer DHIII	LPA_Enhancer_fw19	TGCGCCAGCCGAAGC
Seq. Primer	Enhancer DHIII	LPA_Enhancer_fw7	TACTGCGCTTTTCCGACGGGC
Seq. Primer	Enhancer DHIII	LPA_Enhancer_fw20	CACAGACAAACAAAAAGACAGC
Seq. Primer	Enhancer DHIII	LPA_Enhancer_fw8	CTGAGCAGCCTAACTGGGAGGC
Seq. Primer	Enhancer DHIII	LPA_Enhancer_rv12	CCATATTTCTTGGAGGCTTTGC
Seq. Primer	Enhancer DHIII	LPA_Enhancer_fw9	GAAGTGCTTAAAGGAGCTGATGGAGC
Seq. Primer	Enhancer DHIII	LPA_Enhancer_fw21	CCAAGTTGGAAAACACTCTGC
Seq. Primer	Enhancer DHIII	LPA_Enhancer_fw10	CGGGTTACCTTCAAAGGGAAGCC
Seq. Primer	rs186696265	LPA_rs186696265_rv1	GTTCAGCCCAGGACAGC
Seq. Primer	Promoter	Ch-B_Seq_rv3	GAGATGTCATCTCTGAGGGC
Seq. Primer	Promoter	Ch-B_Seq_rv5	GCACAGGCAAGAGATGAGGC
Seq. Primer	Promoter	Ch-L_Seq_rv1	AAATAGAACGGTTGCAAAATTGC

Seq. Primer	Promoter	Ch-B_Seq_rv6	GGTTGTCTAGTTCAACCTGAGC
Seq. Primer	Promoter	Ch-B_Seq_fw1	TATAATTTATGGGAACTTAGGC
Seq. Primer	Promoter	Ch-L_Seq_rv2	AAAGGAGAGCTACTGATTAATGAGC
Seq. Primer	Promoter	Ch-L_Seq_fw2	CAGGGTACCACCAAGTGGC
Seq. Primer	Promoter	Ch-B_Seq_fw3	TGATGTAATTAGTGAAAGAAGC
Seq. Primer	Promoter	Ch-L_Seq_rv3	AATGCCTCCAGCCAAATGC
Seq. Primer	Promoter	LPAProm_Chen._Fw	ACCTTTGGGGCTGGCTTTCTCAAG
Seq. Primer	Promoter	L-B_Seq_rv1	CCGTTTAAAACAGTATCCAAGC
Seq. Primer	Promoter	LPAProm_Bopp_Fw	TAGGGAAGGAGGTAGAGGGTACAGG
Seq. Primer	Promoter	Ch-B_Seq_fw8	TCCACTTAATTCTACCACTCTTGC
Seq. Primer	Promoter	LPA_unkonv_900_rv	AGCCAGGCCAATGTGGTGAA
Seq. Primer	Promoter	L-B_Seq_fw1	TGTGATCCACCTGTCTTGGC
Seq. Primer	Promoter	Ch-B_Seq_fw9	GGCTGGAGTGCAGTGGC
Seq. Primer	Promoter	Ch-B_Seq_fw6	CACTTATGTTGTCATTTCGGC
Seq. Primer	Promoter	LPA_Prom_rv	GACTTACATGAGAGTAAACGCACCC
Seq. Primer	Promoter	LPA_Bopp.Taramelli_rv2	AAGAACCACTTCCTTATGTTCC
Seq. Primer	Promoter	Ch-B_Seq_fw10	GCTTTCACCACTTCCCAGC
Seq. Primer	Prothrombin rs1799963	rs1799963_fw1	GTCTCATGGGGTGAAGGC
Seq. Primer	ApoB rs781243278	APOB_Ex26_fw2	CTTGCTCTACCAATGCTTTCATACG

* given only for sequencing primers

Supplemental Table VI. SNPs that segregate with the high Lp(a) phenotype in the pedigree following a dominant inheritance pattern.
MAFs are from GnomAD 2.1.1. NFE: Non-finnish Europeans, SAS: South Asian, AFR: African/African American

Chrom.	Position	Rs-ID	Ref	Alt	Gene	Functional class	Consequence	MAF (NFE)	MAF (SAS)	MAF (AFR)
chr4	153332380	rs12644477	T	G	FBXW7	3 prime UTR variant		0.048	0.151	0.189
chr4	155219662	rs17031394	G	C	DCHS2	missense variant	NM_017639.3:p.Thr1480Arg	0.173	0.212	0.174
chr5	150175153	rs2278396	C	T	SMIM3	missense variant	NM_032947.4:p.Arg51Trp	3.91E-04	0.109	4.07E-04
chr5	150480520	rs11960458	C	T	ANXA6	3 prime UTR variant		0.194	0.197	0.125
chr5	150562992	rs555882551	T	C	CCDC69	3 prime UTR variant		0	0.003	0
chr5	150632832	rs1048719	G	A	GM2A	missense variant	NM_000405.4:p.Ala19Thr; NM_001167607.1:p.Ala19Thr	0.030	0.077	0.023
chr5	150647040	rs9324685	C	T	GM2A	3 prime UTR variant		0.030	0.085	0.200
chr5	150647156	rs9324686	T	C	GM2A	synonymous variant, 3 prime UTR variant	NM_001167607.1:p.Ile172Ile	0.029	0.0| 84	0.199
chr6	158517188	.	C	T	SYNJ2	missense variant	NM_003898.3:p.Thr1428Ile; NM_001178088.1:p.Thr1191Ile	NA	NA	NA
chr6	160557643	rs2282143	C	T	SLC22A1	missense variant	NM_003057.2:p.Pro341Leu; NM_153187.1:p.Pro341Leu	0.016	0.075	0.067
chr6	160961137	rs3798220	T	C	LPA	missense variant	NM_005577.2:p.Ile1891Met	0.018	0.004	0.008
chr8	33370261	rs34226219	G	A	TTI2	5 prime UTR variant		0.420	0	0.066
chr9	113261483	rs872665	C	T	SVEP1	missense variant	NM_153366.3:p.Val507Ile	0.253	0.264	0.328
chr18	56963767	rs17769193	C	G	CPLX4	3 prime UTR variant		0.119	0	0.030
chr18	56963852	rs17696504	T	G	CPLX4	3 prime UTR variant		0.119	0	0.030
chr18	56985729	rs12955045	G	A	CPLX4	5 prime UTR, premature start codon gain variant		0.081	0.039	0.023
chr18	57026361	rs33926449	A	G	LMAN1	missense variant	NM_005570.3:p.Val39Ala	0.085	0.039	0.140

Supplemental Table VII. SNPs in candidate genes that were reported to modulate Lp(a) concentrations or act as receptors.
Genes were selected as being putative Lp(a) receptors reviewed in [26] plus *APOB as structural protein of Lp(a), PCSK9* as a known modulator [72,73] and CETP and APOH as new GWAS hits [18] recently described. Only variants that occurred in hyper-Lp(a) individuals but not in controls are shown. If a gene is not listed, no variant was found. No variant segregated perfectly with the genotype. The number of minor alleles found in cases and controls is given, All carriers were heterozygous. For exonic coding variants the consequence on protein level is reported. MAFs are from GnomAD 2.1.1. NFE: Non-Finnish Europeans, SAS: South Asian, AFR: African/African American

**Chrom.**	**Position**	**Rs-ID**	**Ref**	**Alt**	**Gene**	**n alleles cases/ control**	**Functional class**	**Consequence**	**MAF (NFE)**	**MAF (SAS)**	**MAF (AFR)**
chr2	170044661	rs146115458	G	A	LRP2	1/0	synonymous_variant	NM_004525.2:p.Phe3049Phe	2.32E-05	3.27E-05	4.45E-03
chr4	38829702	rs5743816	C	T	TLR6	4/0	missense_variant	NM_006068.4:p.Val465Ile	6.19E-04	0.018	0.077
chr4	38830234	rs5743812	T	C	TLR6	6/0	synonymous_variant	NM_006068.4:p.Thr287Thr	1.01E-04	1.64E-04	0.039
chr4	38830514	rs5743809	A	G	TLR6	6/0	missense_variant	NM_006068.4:p.Leu194Pro	1.02E-04	1.65E-04	0.039
chr4	38830736	rs5743808	A	G	TLR6	4/0	missense_variant	NM_006068.4:p.Ile120Thr	2.87E-03	0.040	0.091
chr12	125263084	rs10396214	G	A	SCARB1	1/0	missense_variant	NM_001082959.1:p.Arg484Trp;<br>NM_005505.4:c.*49C>T	7.33E-03	1.37E-03	1.03E-03
chr12	125348263	rs4238001	C	T	SCARB1	7/0	missense_variant	NM_001082959.1:p.Gly2Ser;<br>NM_005505.4:p.Gly2Ser	0.116	0.069	0.057
chr19	11227554	rs1799898	C	T	LDLR	1/0	synonymous_variant	NM_000527.4:p.Leu575Leu;<br>NM_001195798.1:p.Leu575Leu;<br>NM_001195799.1:p.Leu534Leu;<br>NM_001195800.1:p.Leu407Leu;<br>NM_001195803.1:p.Leu448Leu	0.135	0.133	0.083
chr19	11242044	rs14158	G	A	LDLR	2/0	3_prime_UTR_variant		0.253	0.174	0.173
chr19	45412590	.	C	T	APOE	1/0	3_prime_UTR_variant		NA	NA	NA
chr19	45412600	.	G	C	APOE	1/0	3_prime_UTR_variant		NA	NA	NA
chr2	21225753	rs1042031	C	T	APOB	4/0	missense_variant	NM_000384.2:p.Glu4181Lys	0	6.57E-05	0
chr2	21232175	rs781243278	C	T	APOB	8/0	missense_variant	NM_000384.2:p.Arg2522Gln	1.55E-05	0	8.01E-05
chr2	21249716	rs12691202	C	T	APOB	4/0	missense_variant	NM_000384.2:p.Val730Ile	0.035	3.89E-03	5.65E-03

Supplemental Table VIII. Genotypes of reported regulatory enhancer SNPs of the two alleles of individual II-A2 determined by sequencing *LPA* alleles separated by PFGE.

The individual II-A2 carries the high expressing isoform 21 and a second allele that is not expressed on protein level. The two alleles were separated by Kpn2I-PFGE and the enhancer regions of the two alleles were sequenced. This allows direct determination of which SNP alleles of enhancer SNPs reported by Puckey et al 2003 [28] and Mack et al 2017 [17] are present on each allele.

The G allele of rs9347440 (chr6 : 161106121) increased enhancer activity by 2.5x, the A allele of rs7758766 (chr6 : 161106508) reduced enhancer activity by 30% and the T allele of rs7760010 (chr6 : 161106603) reduces enhancer activity by 40% [28]. AF= frequency of the allele observed on isoform 21 in Europeans (EUR) and South-Asians (SAS), 1000 G project (Ensembl 101).

	**Reference allele**	**Allele 21**	**Unexpressed allele 2 ***	**MAF_{EUR}**	**MAF_{SAS}**
rs9347440	G	G	G	0.436	0.594
rs7758766	C	A	C	0.167	0.090
rs7760010	G	G	G	0.996	1.000
rs186696265	A	A	G	0.013	0.001

* The fragment of the Kpn2I-PFGE is too large to allow precise sizing.

Supplemental Table IX. Genotypes of selected SNPs with known association with isoform or reported, respectively assumed, impact on *LPA* expression

id	Apo(a) isoform 1 (KIV)	Relative expression (%)	Apo(a) isoform 2 (KIV)	Relative expression (%)	rs3798220	rs10455872	rs186696265	rs1800769	rs1853021	rs140570886	PNR	rs7758766	rs9347440	rs7760010
II-A1	**24**	37	**33**	63	TT	AA	GG	AG	CC	TT	8/10	CA	GA	GG
III-A4	**25**	100	**-**	0	TT	AA	GG	GG	CC	TT	8/8	CC	GA	GG
III-A1	**21**	77	**33**	23	CT	AA	AG	AA	CC	TC	8/8	CA	GA	GG
III-A2	**21**	77	**33**	23	CT	AA	AG	AA	CC	TC	8/8	CA	GA	GG
III-A3	**21**	77	**33**	23	CT	AA	AG	AA	CC	TC	8/8	CA	GA	GG
II-C2	**21**	100	**-**	0	CT	AA	AG	AG	CT	TC	8/9	CA	GG	GG
II-B1	**21**	99	**28**	1	CT	AA	AG	AG	CC	TC	8/8	CA	GA	GG
II-A2	**21**	100	**-**	0	CT	AA	AG	AG	CT	TC	8/9	CA	GG	GG
II-D1	**21**	100	**-**	0	CT	AA	AG	AG	CT	TC	8/9	CA	GG	GG
III-A5	**21**	95	**24**	5	CT	AA	AG	AG	CC	TC	8/10	AA	GG	GG
II-B2	**30**	89	**38**	11	TT	AA	GG	AG	CC	TT	9/10	CC	GA	GG
III-B1	**21**	80	**38**	20	CT	AA	AG	AA	CC	TC	8/9	CA	GG	GG
III-B3	**28**	2	**30**	98	TT	AA	GG	GG	CC	TT	8/10	CC	AA	GG
III-B4	**21**	70	**30**	30	CT	AA	AG	AG	CC	TC	8/10	CA	GA	GG
III-B5	**21**	75	**30**	25	CT	AA	AG	AG	CC	TC	8/10	CA	GA	GG
II-C1	**15**	47	**20**	53	TT	AG	GG	GG	CC	TT	8/8	CA	GA	GG
III-C1	**15**	33	**21**	67	CT	AA	AG	AG	CC	TC	8/8	AA	GG	GG
III-C2	**20**	nd	**21**	nd	CT	AG	AG	AG	CC	TC	8/8	CA	GA	GG
III-C3	**15**	100	**-**	0	TT	AA	GG	GG	CT	TT	8/9	CA	GG	GG
III-C4	**15**	33	**21**	67	CT	AA	AG	AG	CC	TC	8/8	AA	GG	GG
II-D2	**21**	100	**-**	0	-	-	-	-	-	-	-	-	-	-
III-A6	**24**	100	**-**	0	-	-	-	-	-	-	-	-	-	-

Rs3798220: I4399M; rs186696265 and rs140570886: top hits of Mack et al [17]; PNR: pentanucleotide repeat polymorphism in the *LPA* promoter; rs7758766, rs9347440, rs7760010: functional enhancer SNPs reported by Puckey et al [28]; rs1800769 and rs1853021: functional polymorphisms traditionally named +121 G/A and +93 C/T. The former has been associated with increased transcription, while the latter has been reported to reduce *LPA* translation. Rs140570886 was determined by imputation, all other variants were determined by sequencing or genotyping. The G allele of rs9347440 (chr6 : 161106121) increased enhancer activity by 2.5x, the A allele of rs7758766 (chr6 : 161106508) reduced enhancer activity by 30% and the T allele of rs7760010 (chr6 : 161106603) reduces enhancer activity by 40%.

Supplemental Table X. Prothrombin G20210A (rs1799963) genotypes in the pedigree.

ID	CVD	Prothrombin G20210A
II_A1	no	GG
III_A4	no	GG
III_A1	no	AG
III_A2	no	GG
III_A3	yes	AG
II_C2	no	GG
II_B1	yes	GG
II_A2	no	AG
II_D1	yes	AG
III_A5	no	AG
II_B2	no	GG
III_B1	yes	GG
III_B3	no	GG
III_B4	no	GG
III_B5	no	GG
II_C1	yes	GG
III_C1	no	GG
III_C2	no	GG
III_C3	no	GG
III_C4	yes	GG
II_D2	no	NA

SUPPLEMENTAL FIGURES

Supplemental Figure I. LDLR expression as well as LDL and Lp(a) cellular uptake are similar in controls and patients with hyperlipoproteinemi(a)emia. PBMCs were plated for 24h in serum deprived medium with or without mevastatin (10 µg/mL) and supplemented or not for the last 4h of the incubation with rPCSK9 (600 ng/mL) with or without alirocumab (19.2 µg/mL) prior to flow cytometry analysis. (**A**) Cell surface LDLR expression, (**B**) LDL-bodipy uptake, and (**C**) Lp(a)-bodipy uptake in primary lymphocytes isolated from three control normolipoprotein(a)emic family members, four hyperlipoprotein(a)emic family members, and one LDLR-negative homozygous FH patient. Data are expressed in ΔMFI. Histograms represent mean ± SEM of a minimum of three independent experiments performed in duplicates. *, p<0.05.

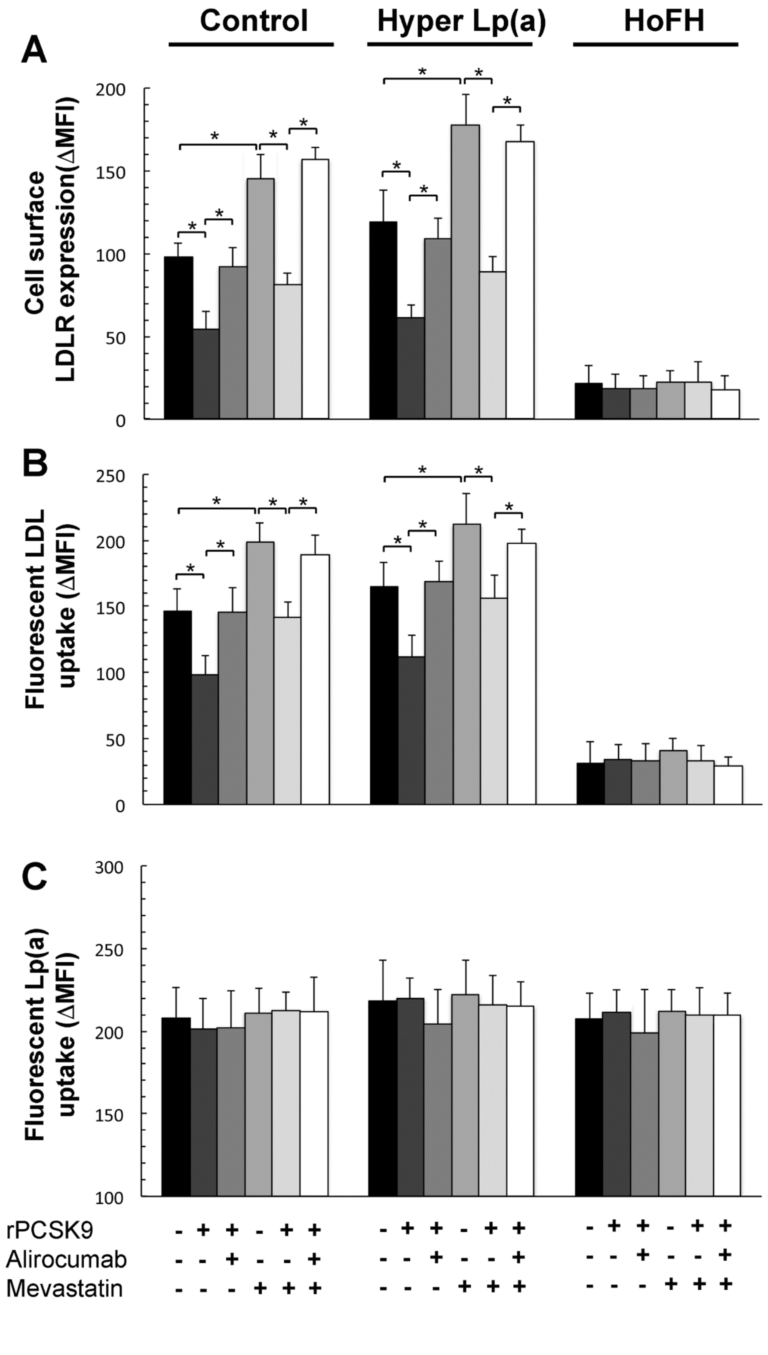

Supplemental Figure II. Ancestry estimation using LASER.

Location of the pedigree samples in the reference principal component analysis (PCA) space generated by the LASER program. The reference PCA is constructed using 632,958 autosomal SNPs of 938 unrelated individuals from the Human Genome Diversity Project [67].

Most samples of the pedigree cluster close to Central/South-Asians (C/S Asia), Middle Eastern and European samples, with two samples showing signs of admixture from African groups. This is in line with the population history of La Réunion.

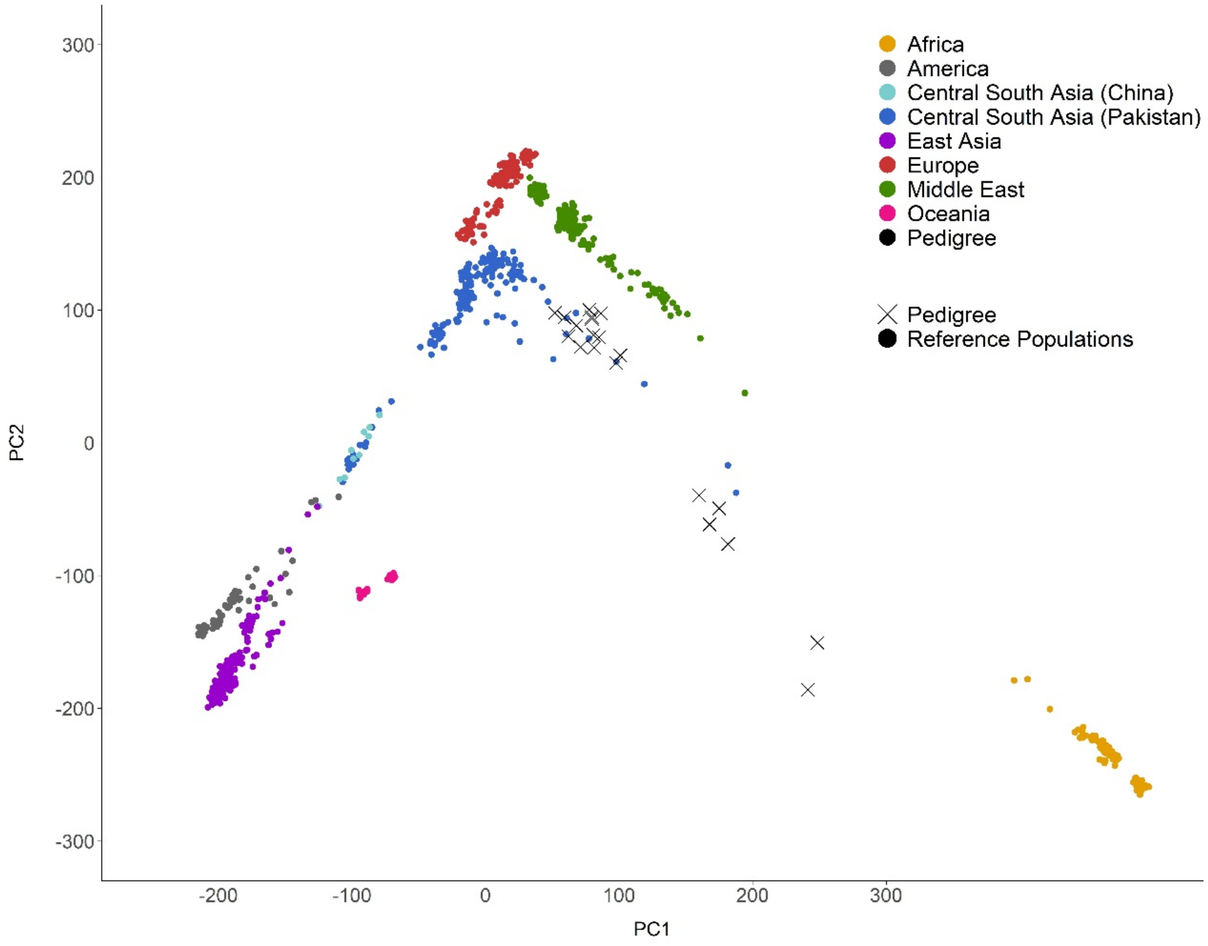

Supplemental Figure III. Distribution of the genetic risk score (GRS) for Lp(a) in the 1000G super populations – **A**, all 1000G populations. **B**, EUR and SAS only (populations most similar to the pedigree individuals, see Supplemental Figure 2). **C**, Distribution of the GRS for Lp(a) in KORA F4 (for comparison). AFR: Africans, AMR: Admixed Americans, EAS: East Asians, EUR: Europeans, SAS: South-Asians.

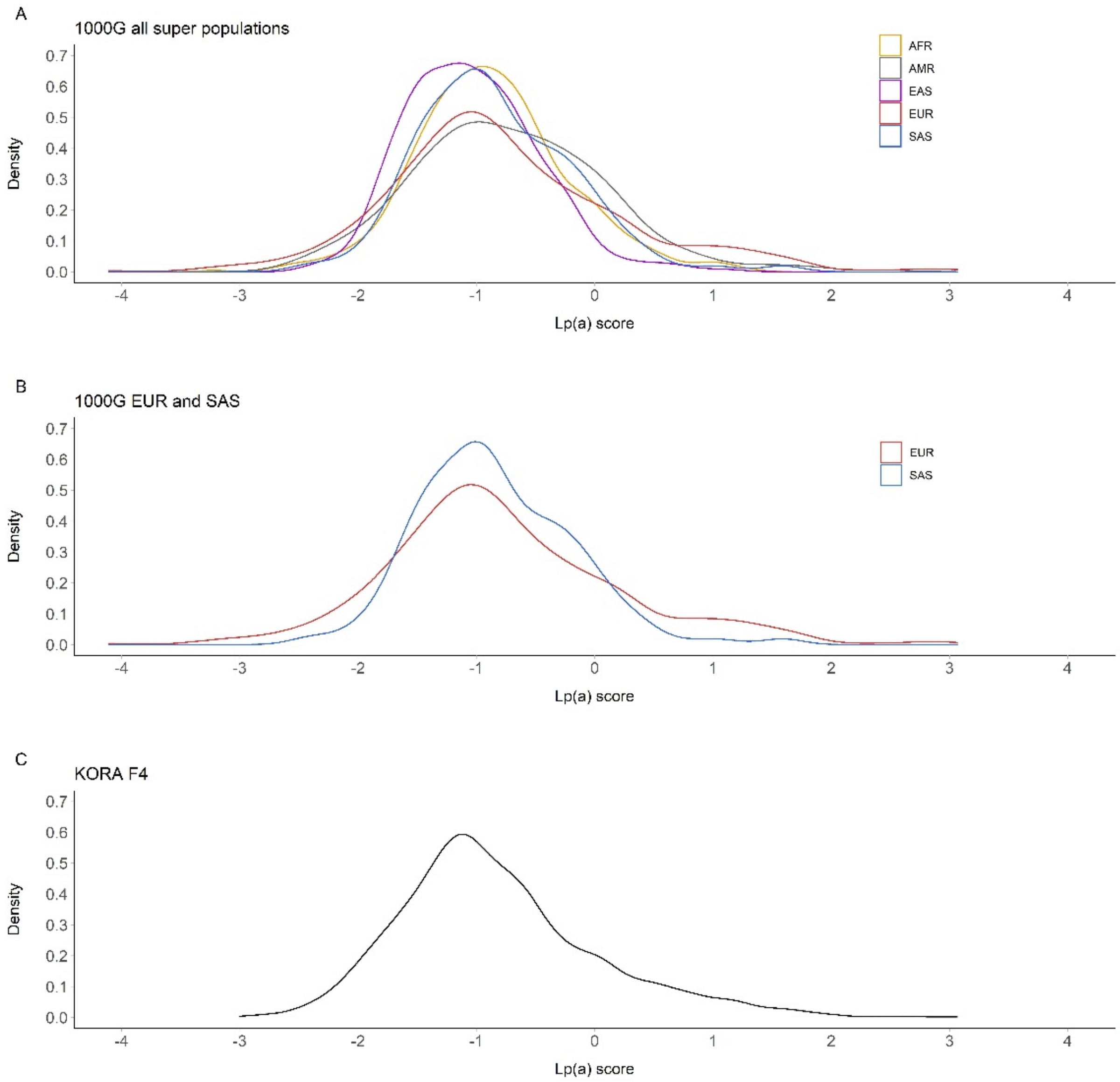

Supplemental Figure IV. GRS distribution for CAD in the 1000G populations – GRS distribution in the European (**Panel A**) and South-Asian (**Panel B**) 1000G super populations with the location of the family members shown by the dots below. Family members who had CVD are depicted in orange, the others in black. Distribution of the CAD GRS in all 1000G super populations is shown in **Panel C**. Colors indicate the bottom/top 5th (dark blue) and 2.5th (light blue) percentiles. AFR: Africans, AMR: Admixed Americans, EAS: East Asians, EUR: Europeans, SAS: South-Asians.

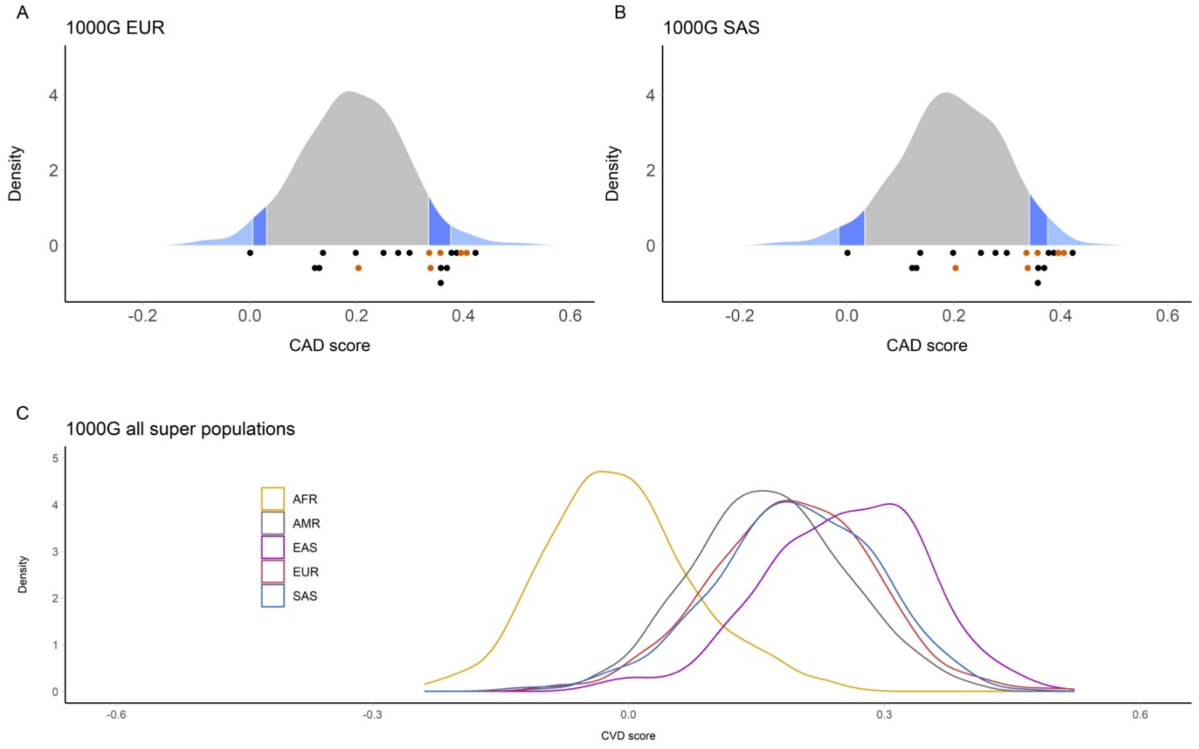

Supplemental Figure V. GRS distribution for Venous Thrombo-Embolism (VTE) indicating the bottom/top 5th (dark blue) and 2.5th (light blue) percentiles of the KORA F4 (**Panel A**) the general 1000G Europeans (**Panel B**) and South Asians (**Panel C**). Distribution of the VTE GRS among 1000G super populations of different ancestries (**Panel D**). VTE GRS of family members who had CVD are depicted in orange, the others in black below each chart – AFR: Africans, AMR: Admixed Americans, EAS: East Asians, EUR: Europeans, SAS: South Asians.

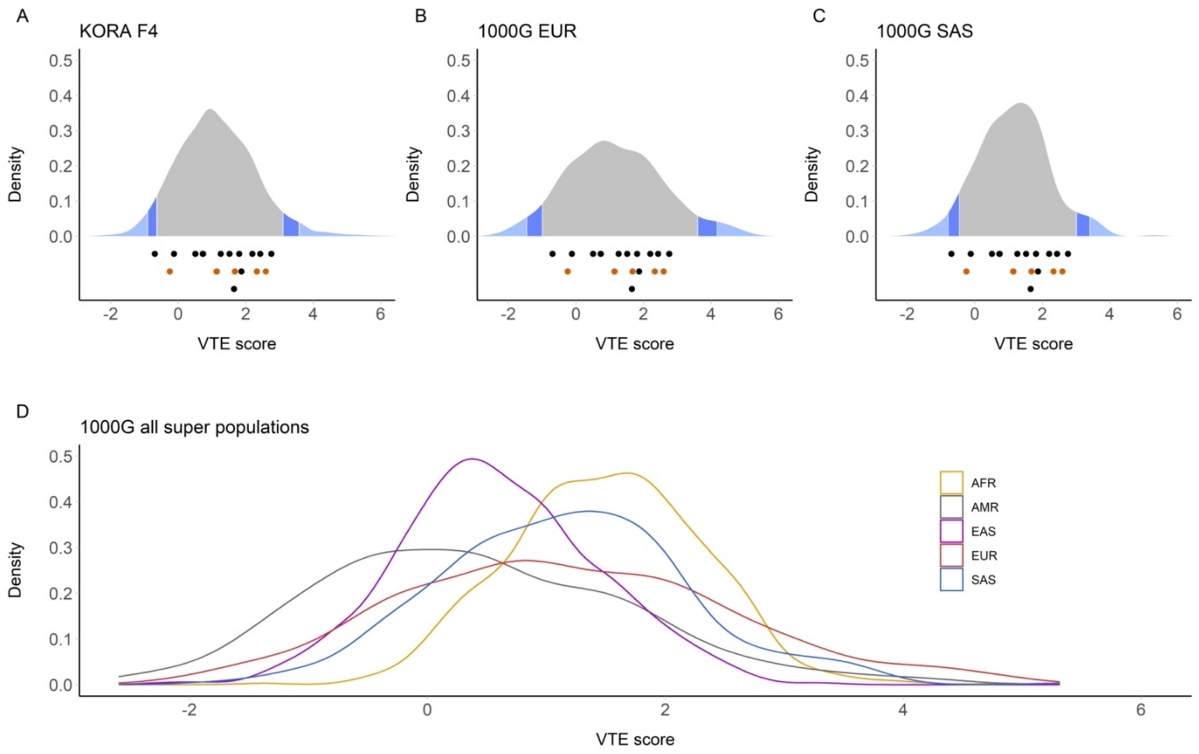

Supplement: Supplementary file 1 [file hcg-15-e003489-s001.pdf]
